# Frontiers in Antibody–Drug Conjugates: Mechanisms, Design Innovations, and Clinical Applications in Targeted Cancer Therapy

**DOI:** 10.3390/ph19020324

**Published:** 2026-02-15

**Authors:** Xinghan Li, Jiaming Liu, Yitong Meng, Jun Li, Jieling Zhao, Dequan Liu, Xiaodong Zhang

**Affiliations:** 1 Department of Stomatology, General Hospital of Northern Theater Command, No. 83, Wenhua Road, Shenhe District, Shenyang 110016, China; lxh950601@163.com (X.L.); mengyitong6162599@foxmail.com (Y.M.); 17790972112@163.com (J.L.); zljie20120316@163.com (J.Z.); 2 Neurosurgery, General Hospital of Northern Theater Command, No. 83, Wenhua Road, Shenhe District, Shenyang 110016, China; liujiaming041128@126.com; 3 Department of Urology, the First Affiliated Hospital of Dalian Medical University, Dalian 116011, China

**Keywords:** antibody–drug conjugates, cytotoxic agents, mechanisms of action, payload design, clinical advancements

## Abstract

Antibody–drug conjugates (ADCs) represent a transformative class of targeted therapies designed to deliver potent cytotoxic agents specifically to tumor cells, minimizing systemic toxicity. This review provides a comprehensive overview of ADCs, detailing their mechanisms of action, design strategies, and clinical advancements. ADCs utilize monoclonal antibodies to selectively bind tumor-associated antigens, enabling the precise delivery of toxic payloads to cancer cells. The review explores the critical components of ADCs, including the antibody, linker, and payload, and highlights how these elements can be optimized to improve efficacy and minimize off-target effects. We examine the evolution of ADC design from early constructs to the latest innovations and the development of novel payloads that extend therapeutic possibilities beyond traditional cytotoxic agents. Additionally, we discuss the clinical success of ADCs, with examples from approved therapies such as gemtuzumab ozogamicin, brentuximab vedotin, and trastuzumab emtansine, which have redefined the treatment landscape for various cancers. Despite their success, ADCs face challenges such as tumor heterogeneity, resistance mechanisms, and toxicity, which are actively being addressed through ongoing research. The review concludes with an outlook on the future of ADCs, highlighting emerging strategies in conjugation technology, payload design, and combination therapies that are poised to enhance their therapeutic potential across oncology and other disease areas.

## 1. Introduction

In recent decades, the landscape of cancer treatment has undergone a significant transformation, moving away from traditional chemotherapy and toward more targeted and specific therapies. Chemotherapy, which was the primary treatment option from the early 20th century, provided substantial clinical benefits but was associated with substantial systemic toxicity [1]. This non-specific treatment approach affected not only cancerous cells but also normal proliferating cells in tissues such as bone marrow, gastrointestinal epithelium, and hair follicles, leading to severe side effects [1]. In response to these limitations, the introduction of monoclonal antibodies (mAbs) and targeted therapies, such as rituximab (anti-CD20) and trastuzumab (anti-HER2), revolutionized clinical oncology by enabling the selective targeting of specific cancer antigens, thereby minimizing off-target damage. More recently, immune checkpoint inhibitors (ICIs) and bispecific antibodies have further advanced cancer therapy, enhancing the immune system’s ability to recognize and destroy cancer cells.

Antibody–drug conjugates (ADCs) are engineered biologics that aim to concentrate highly potent cytotoxic agents within tumor cells while minimizing systemic exposure [2]. Compared with small-molecule targeted inhibitors such as kinase inhibitors, which require that the tumor depend on a specific oncogenic signaling pathway and are prone to resistance via pathway rewiring, ADCs do not need to inhibit a driver pathway: they only require that the target antigen be expressed on the cell surface and internalized, after which the payload enforces direct cytotoxic stress [3]. Further, ADCs with cleavable linkers and membrane-permeable payloads can exert a “bystander effect,” diffusing into adjacent tumor cells with lower or heterogeneous antigen expression and thereby addressing spatial heterogeneity in solid tumors [3]; ADCs convert that same targeting scaffold into a delivery vehicle for a lethal intracellular payload, enabling direct tumor cell killing even when immune effector function is weak or when the target antigen is not itself an oncogenic driver [4]. Early ADCs such as gemtuzumab ozogamicin (anti-CD33), brentuximab vedotin (anti-CD30), and HER2-directed conjugates like trastuzumab emtansine (T-DM1) and trastuzumab deruxtecan (T-DXd) helped move the modality from niche salvage therapy to a backbone option, demonstrating that even heavily pretreated, otherwise refractory disease could be controlled by delivering lethal payloads directly into antigen-expressing tumor cells [5,6,7,8]. For instance, in 2018, the phase 3 ECHELON-1 trial enrolled 1334 previously untreated patients with stage III/IV classic Hodgkin lymphoma [6]. At a median follow-up of 24.6 months, Brentuximab Vedotin (BV) plus doxorubicin, vinblastine, and dacarbazine (A + AVD) produced a higher 2-year modified progression-free survival rate than doxorubicin, bleomycin, vinblastine, and dacarbazine (ABVD), supporting A + AVD as a more effective frontline option for advanced-stage disease [6]. Overall, ADCs decouple the targeting of antibodies from the lethality of the payload, opening up therapeutic space in heterogeneous tumors and in tumors resistant to prior therapies.

## 2. The Evolution of ADCs

The therapeutic concept of antibody–drug conjugates (ADCs) was first proposed in the 1970s as an explicit attempt to turn antibodies into selective delivery vehicles for highly toxic agents. The idea emerged soon after Köhler and Milstein developed hybridoma technology in 1975 [9,10]. Once researchers realized they could generate antibodies that reliably recognized a tumor-associated antigen, they began asking a simple question: instead of giving a non-specific chemotherapeutic drug to the whole body, could you chemically “bolt” a toxic payload onto an antibody and use that antibody to carry the toxin directly and specifically to cancer cells [11]. Early constructs, built by chemically coupling murine antibodies to protein toxins like ricin A chain or diphtheria fragments, demonstrated proof-of-principle in vitro. For instance, in 1978, using erythrocyte ghosts to deliver a defined number of diphtheria toxin fragment A molecules into otherwise resistant mouse L cells, Yamaizumi et al. demonstrated that introduction of even a single molecule of fragment A was sufficient to abrogate colony-forming ability, to kill an individual cell [12].

The first clinical wave in the 1990s–early 2000s addressed some of these limitations through the use of humanized or chimeric antibodies and small-molecule warheads [13,14]. This led to the approval of gemtuzumab ozogamicin in 2000 for CD33+ acute myeloid leukemia, which established that ADCs could achieve therapeutic efficacy in humans [13,15]. In phase I/II studies in relapsed acute myeloid leukemia (AML) it demonstrated an overall response rate of approximately 30% (including complete responses with incomplete platelet recovery) [15]. It also highlighted critical liabilities such as premature linker cleavage, systemic toxicity (notably hepatotoxicity), and a narrow therapeutic index [15]. This experience defined the first major design lesson for the field: stability in circulation is essential.

**Figure 1:**
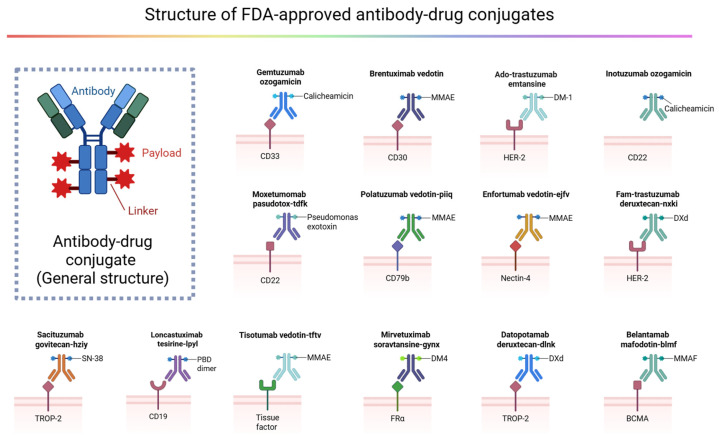
Structure of ADCs. CD33, Calicheamicin; CD30, MMAE (auristatin E); HER2, DM1 (maytansinoid); CD22, Calicheamicin; CD22, Pseudomonas exotoxin (recombinant immunotoxin); CD79b, MMAE; Nectin-4, MMAE; HER2, DXd (exatecan derivative; topo-I inhibitor); TROP2, SN-38 (topoisomerase I inhibitor); CD19, PBD dimer (SG3199); Tissue factor (TF), MMAE; FRα, DM4 (maytansinoid); TROP2, DXd (exatecan derivative; topo-I inhibitor); BCMA, MMAF (auristatin F). Created in BioRender. Liu, D. (2026) https://BioRender.com/govuguf (accessed on 9 February 2026).

Subsequent generations of ADCs have refined each core element—antibody, linker, and payload—into a clinically reliable delivery platform (Figure 1). Second-generation agents such as BV (targeting CD30, with a protease-cleavable dipeptide linker and an auristatin payload) and trastuzumab emtansine/T-DM1 (targeting HER2, with a non-cleavable thioether linker and a maytansinoid payload) demonstrated that rational linker design could either enable controlled intracellular drug release through tumor-associated proteolysis or enforce exceptional plasma stability by requiring full lysosomal degradation of the conjugate [16,17]. In the phase 3 EMILIA trial of 991 patients with previously treated HER2-positive advanced breast cancer, T-DM1 significantly improved progression-free survival (9.6 vs. 6.4 months; HR 0.65; *p* < 0.001) and overall survival (30.9 vs. 25.1 months; HR 0.68; *p* < 0.001) compared with lapatinib plus capecitabine, with a higher objective response rate and a more favorable overall toxicity profile [17]. These advances, together with the selection of antigens enriched on tumor cells and capable of efficient internalization, helped establish ADCs as standard-of-care options rather than experimental salvage therapies. Parallel improvements in conjugation chemistry—moving from heterogeneous, random lysine/cysteine coupling toward site-specific conjugation—enabled consistent drug-to-antibody ratios (DAR), cleaner pharmacokinetics, and manufacturability at scale. Building on the clinical success of agents such as BV (Adcetris) and trastuzumab emtansine (Kadcyla), current efforts in conjugation chemistry, linker stability/cleavability, and site-specific conjugation to generate more homogeneous ADCs are accelerating the field [18].

Current and emerging ADC designs push beyond simple “antibody plus toxin” toward modular, programmable delivery systems. Modern development priorities include expanding the therapeutic index by tightening tumor selectivity (for example, through bispecific or biparatopic targeting); bispecific antibody–drug conjugates (BsADCs) are an emerging class of therapeutics that integrate the dual-targeting capabilities of bispecific antibodies with the targeted cytotoxic delivery of antibody–drug conjugates (ADCs), with the goal of overcoming key limitations of current ADCs such as suboptimal internalization, off-target toxicity, and acquired resistance, with approximately ten BsADCs already in clinical trials [19]. ADCs have ushered in a new era of targeted cancer therapy, with 14 drugs currently approved for clinical use by the U.S. Food and Drug Administration (FDA) (Table 1). Although oncology remains the dominant application space, the same logic—deliver an otherwise intolerable small molecule specifically to a pathogenic cell population—is now being explored in areas such as autoimmunity, fibrosis, and infectious reservoirs [20]. Collectively, the trajectory of ADCs can be understood as the progressive transformation of monoclonal antibodies from passive targeting agents into precision delivery vehicles for highly controlled intracellular pharmacology.

## 3. Mechanism of Action of ADCs

**Figure 2:**
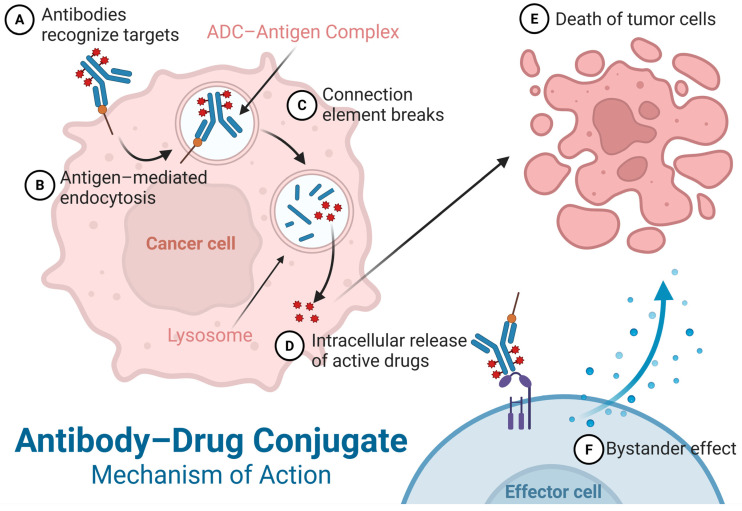
Canonical ADC pathway. The action steps of ADC can be roughly divided into six steps, namely antibody recognition of the target, antigen-mediated endocytosis, stability and cleavage of the linker, intracellular release of the active drug, and killing of the target cell. In addition, it also includes the bystander effect, that is, the released drug molecules can kill adjacent tumor cells without antigen expression. ADCs, antibody–drug conjugates. Created in BioRender. Liu, D. (2026) https://BioRender.com/x54ki8u (accessed on 9 February 2026).

The mechanism of action of ADCs includes antibody recognition of the target, antigen-mediated internalization, linker stability and cleavage, intracellular release of the active drug, and target cell killing. In brief, the antibody recognizes and binds with high affinity to specific antigens on the surface of tumor cells, forming an ADC–antigen complex. This complex is then internalized into the cell via receptor-mediated endocytosis and transported to degradation pathways such as the lysosome. Within the lysosome, the linker in the complex is cleaved, triggered by specific enzymes or the acidic environment, releasing the active cytotoxic payload. This payload exerts cytotoxic effects such as growth inhibition or DNA damage, ultimately inducing tumor cell apoptosis [3,21,22]. Furthermore, some ADC designs incorporate a bystander effect, whereby the released drug molecules can kill adjacent tumor cells that do not express the target antigen, further enhancing antitumor activity [23] (Figure 2).

### 3.1. Targeted Recognition and Antibody Types in ADCs

#### 3.1.1. Target Specificity of Monoclonal Antibodies and Advantages of Multi-Target ADCs

**Figure 3:**
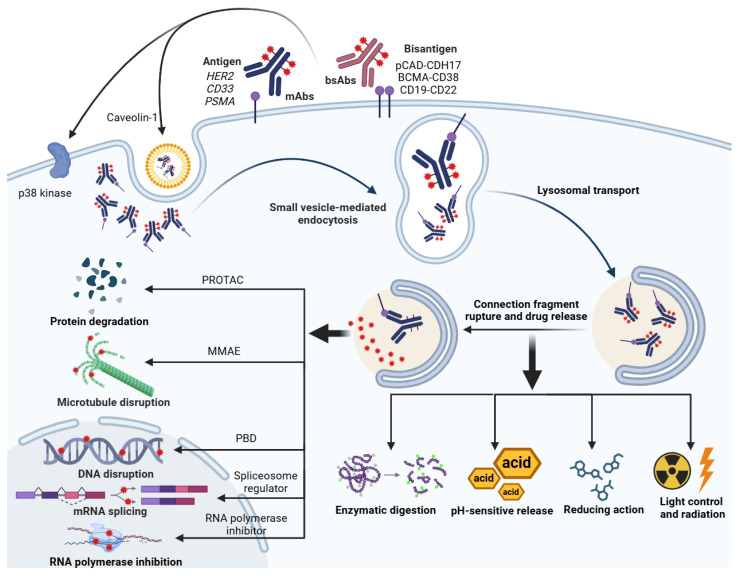
Expanded ADC mechanisms. The mechanism of action of ADCs involves mAbs recognizing specific antigens on the surface of tumor cells, such as HER2, CD33, PSMA, etc., to precisely target tumor cells. bsAbs enhance the tumor cell recognition ability and endocytosis of ADCs by simultaneously recognizing two different antigens or different epitopes of the same antigen. The entry of ADCs into cells through lipid raft-mediated endocytosis depends on the expression of caveolin-1 protein, while the p38 kinase-mediated atypical endocytosis pathway does not rely on the tyrosine kinase activity of the receptor. Subsequently, the therapeutic effect of ADC drugs based on the endosome–lysosome pathway is crucial. During this process, the classical caveolae-mediated endocytosis pathway is often involved. Enzymatic release and pH-sensitive release are the most widely used drug release methods in ADCs. In addition, there are also novel triggering mechanisms such as reduction environment-triggered release, light-controlled release, and radiation-triggered release. Finally, the mechanism of action of cytotoxic payloads includes microtubule inhibition, DNA damage, spliceosome regulation, blocking RNA synthesis, and selective degradation of target proteins. ADC, antibody–drug conjugates; HER2, human epidermal growth factor receptor 2; CD33, Sialic acid-binding Ig-like lectin 33; PSMA, Prostate-specific membrane antigen. Created in BioRender. Liu, D. (2026) https://BioRender.com/hsigrlu (accessed on 9 February 2026).

Monoclonal antibodies (mAbs), due to their high specificity and affinity for tumor cells, serve as the core component of ADCs for achieving precise targeted delivery. Specifically, mAbs recognize tumor cell surface-specific antigens, such as HER2, CD33, and PSMA, enabling precise localization to tumor cells, thereby maximizing the therapeutic selectivity and potency of the ADC. For instance, HER2, a key member of the human epidermal growth factor receptor family, is overexpressed in various tumors like breast and gastric cancers, making it a classic target for ADCs [24,25]. Additionally, CD33 is highly expressed in acute myeloid leukemia and represents another important target. The high-affinity binding of mAbs to these tumor-associated antigens allows ADCs to specifically recognize and bind tumor cells, followed by internalization and intracellular drug release for killing (Figure 3).

However, heterogeneity in target expression is a critical factor affecting the targeting efficiency of ADCs. The expression of target antigens on tumor cells varies spatially and temporally, leading to differential binding capacity of the ADC across different regions of the same tumor or on different cells. This can limit the efficacy of traditional single-target ADC therapies when tumor cell surface antigen expression is heterogeneous or low, and can easily lead to drug resistance [26]. Moreover, low-level expression of the target in normal tissues can also lead to non-specific uptake of the ADC, causing “on-target, off-tumor” toxicity and limiting ADC safety. In this context, bispecific antibodies (bsAbs), as an innovative antibody format, enhance the tumor cell recognition and internalization capabilities of ADCs by simultaneously recognizing two different antigens or different epitopes on the same antigen [26,27]. Currently, various bispecific antibodies and ADCs have entered clinical development stages, with some already approved by the FDA for cancer treatment [28]. For example, a bispecific ADC targeting pCAD and CDH17 can selectively kill colorectal cancer cells co-expressing both antigens, significantly improving tumor specificity and reducing toxicity to normal cells expressing only one antigen [29]. Similarly, a dual-targeting strategy for BCMA and CD38 in multiple myeloma leverages the high expression of these antigens on malignant plasma cells, overcoming issues of antigen downregulation and resistance associated with single-antigen targeting. Likewise, dual-targeting ADC designs for CD19 and CD22 in acute lymphoblastic leukemia have shown significant efficacy, reducing the potential for cancer cell immune escape [30,31] (Figure 3).

#### 3.1.2. Antibody Conjugation Sites and Methods

The performance of ADCs heavily depends on the choice of conjugation sites and the methods used to link the antibody and the drug. Early ADCs often employed random conjugation techniques, such as non-specific modification of lysine or cysteine residues, resulting in products with high heterogeneity, limited stability, and efficacy. In recent years, with advancements in genetic engineering and chemoenzymatic catalysis, site-specific conjugation has become a key direction for antibody modification, significantly improving the homogeneity, stability, and therapeutic index of ADCs.

Genetic engineering methods, such as incorporating unnatural amino acids or mutating specific amino acids, enable precise modification at specific sites. For instance, introducing specific cysteine residues into antibodies allows for site-specific conjugation using selective reducing agents while maintaining the native disulfide bond structure, thereby generating homogeneous ADC molecules and avoiding antibody aggregation issues associated with traditional reduction-oxidation steps [32,33]. Secondly, amino acid metabolic engineering is an important strategy for precise drug loading. For example, utilizing MTG for enzyme-catalyzed conjugation at specific glutamine residues on the antibody allows for highly controlled conjugation sites, generating ADCs with good in vivo stability and efficacy [34,35]. Furthermore, antibody modification technology mediated by lipoic acid ligase, which selectively links short-chain fatty acids to specific lysine residues on the antibody, has been studied. Molecular dynamics simulations have been used to reveal the influence of the local amino acid environment on enzyme catalytic selectivity, further guiding the rational design of conjugation sites [35,36,37].

Notably, the selection of antibody conjugation sites also directly impacts the pharmacokinetics and intracellular killing efficacy of ADCs. Studies have shown that conjugating cytotoxic drugs to specific sites in the Fc region of the antibody (such as Lys248, Lys288, or the C’E loop) can significantly improve ADC stability, circulation half-life, and therapeutic effect [38,39,40]. For example, ADCs generated using AJICAP technology, which employs Fc-affinity peptide-mediated site-specific conjugation, demonstrate a superior therapeutic index and lower immunogenicity [41,42]. Additionally, multifunctional conjugation technologies allow for dual or multiple drug loadings on the same antibody, utilizing different conjugation sites and chemical reactions to achieve synergistic or complementary combination therapies, enhancing antitumor activity and overcoming resistance [43,44,45]. Strategies combining enzymatic catalysis, chemical conjugation, and protein engineering enable specific conjugation of antibodies to proteins or oligonucleotides, broadening the application scope of ADCs [46,47].

### 3.2. Intracellular Internalization and Trafficking Mechanisms of ADCs

#### 3.2.1. Receptor-Mediated Endocytic Pathways

A key step for ADCs to exert their antitumor effect is entering the cell via receptor-mediated endocytosis on the tumor cell surface. Specifically, the ADC first binds to the target antigen highly expressed on the tumor cell surface. Subsequently, through receptor-mediated endocytic mechanisms, the ADC is internalized into the cell, effectively delivering the cytotoxic drug intracellularly for precise killing. This internalization process primarily includes clathrin-mediated endocytosis and non-clathrin-mediated endocytosis [48,49].

Research indicates that the target antigen bound by the ADC determines the specific type of endocytic pathway. For example, HER2-targeting ADCs (such as T-DM1) in HER2-positive tumor cells primarily enter the cell via lipid raft-mediated endocytosis, a process dependent on caveolin-1 protein expression; disruption of lipid raft structure significantly inhibits ADC internalization and degradation [50]. Another typical example is EGFR-targeting ADCs; the internalization of EGFR-ADC can occur rapidly via a p38 kinase-mediated atypical endocytic pathway, which is independent of the receptor’s tyrosine kinase activity [51]. Furthermore, the efficiency of receptor-mediated endocytosis is regulated by multiple factors, including receptor expression levels, receptor internalization kinetics, the cell membrane microenvironment, and the structural properties of the ADC itself. For instance, cIRCR201-dPBD, a novel pyrrolobenzodiazepine dimer ADC precisely targeting c-Met overexpressing tumor cells, shows internalization efficiency highly dependent on c-Met expression levels on the tumor cell surface [52] (Figure 3).

#### 3.2.2. Intracellular Complex-Lysosome Trafficking

After internalization into tumor cells, ADCs are transported to endosomes and further trafficked to lysosomes to complete the drug release process. Specifically, the ADC first binds to the target antigen on the tumor cell surface via its specific antibody portion, forming an ADC–antigen complex. This complex is then internalized, forming early endosomes, and undergoes multistage transport ultimately reaching the lysosomes. The acidic environment and specific enzymatic actions within the lysosomes trigger linker cleavage, releasing the cytotoxic payload and inducing tumor cell death [22]. Therefore, the integrity and efficiency of the endosome–lysosome pathway are crucial for the therapeutic efficacy of ADC drugs. This process often involves the classical caveolae-mediated endocytic pathway, where caveolae are flask-shaped invaginations of the plasma membrane (Figure 2). Taking RC48-ADC as an example, after binding HER2, it is effectively internalized into lysosomes via caveolae. The linker is enzymatically cleaved within the lysosome, releasing monomethyl auristatin E (MMAE) to achieve targeted killing [49,53]. Additionally, ADCs targeting different antigens exhibit differences in internalization efficiency and endosomal trafficking processes, affecting the final drug release and antitumor activity [49].

### 3.3. Drug Release Mechanisms

The linker, as a key component of the ADC, serves the dual function of ensuring ADC stability in circulation and enabling effective release of the cytotoxic drug after targeted delivery. Linkers are primarily classified into two categories: cleavable linkers and non-cleavable linkers. Cleavable linkers are typically designed to break under the acidic conditions of the lysosome or through enzymatic action, releasing the active drug. These include enzyme-cleavable linkers and acid-labile linkers, such as the valine-citrulline linker used in RC48-ADC [53]. Non-cleavable linkers release the drug only after antibody degradation, at which point the drug remains attached to the linker residue. This can affect the drug’s cell-penetrating ability and bystander effect [54]. Common trigger mechanisms for linker cleavage include enzymatic release, pH-sensitive release, and reduction-triggered release.

Enzymatic release is one of the most widely used drug release mechanisms in ADCs. The core design principle of enzyme-cleavable linkers is to leverage the high expression of specific enzymes within tumor cells to achieve specific intracellular cleavage. They are typically designed to contain enzyme-sensitive peptide sequences, such as Val-Cit, Phe-Gly, Val-Ala-Gly, etc., utilizing lysosomal enzymes to mediate linker cleavage and enable precise drug release. For instance, studies show that linkers containing the Val-Ala-Gly tripeptide sequence can efficiently release drugs in the presence of cathepsin B [55]. Furthermore, some novel linkers have been developed to respond to the activity of specific enzymes in the tumor microenvironment. For example, human neutrophil elastase-sensitive linkers enable a dual drug release mechanism for ADCs both inside and outside tumor cells, enhancing antitumor activity [56]. Gly-Pro-Leu-Gly (GPLG) has been found to be cleaved faster by Cathepsin B and possesses superior stability, showing potential as a next-generation enzyme-cleavable linker [57] (Figure 2).

The pH-sensitive release mechanism exploits the acidic environmental differences within tumor cells, causing the linker to break under low pH conditions and release the active drug. Acid-labile linkers are typically designed to hydrolyze in acidic environments, releasing the drug. Acid-sensitive linkers such as hydrazone, cyclic acetal, and other structures maintain plasma stability while utilizing intracellular acidic conditions for drug release [58]. One study, using fluorescence tracing technology, revealed the localization and drug release kinetics of ADCs in the acidic endosome–lysosome pathway, confirming the high efficiency of pH-triggered drug release [59]. For example, pH-sensitive phosphoramidate linkers have been used to construct ADCs targeting prostate cancer, enabling efficient drug release in acidic environments and promoting intracellular drug accumulation [60]. However, acid-labile linkers are susceptible to the surrounding amino acid environment; certain specific sites on the antibody, such as residues near Lys-207, may promote their hydrolysis, leading to reduced ADC stability [61]. Site-directed mutagenesis of the conjugation site amino acids or extending the linker length can effectively improve the stability of acid-labile linkers [61] (Figure 2).

Besides the two classic drug release mechanisms mentioned above, reduction-triggered release primarily utilizes the higher concentration of reducing molecules (such as glutathione, GSH) inside cells compared to the extracellular environment. Linkers containing reducible disulfide bonds or thioether groups are designed to be cleaved by these reducing molecules upon entering the cell, thereby releasing the active drug (Figure 3). For instance, glutathione-selective phenoxysilane linkers have achieved effective drug release in cells with high glutathione levels, improving the therapeutic window [62]. To address issues of insufficient stability or suboptimal release efficiency in traditional linkers, novel trigger mechanisms such as photo-controlled release and radiation-triggered release are gradually being incorporated into ADC design (Figure 2). Strategies using near-infrared light irradiation to induce linker cleavage and drug release have successfully achieved drug release in non-specific tumor cells, enhancing the bystander effect of treatment [63]. Similarly, using X-ray radiation to induce linker cleavage enables spatial control over local drug release, significantly improving the tumor-killing effect of ADCs [64]. For non-cleavable linkers, the drug is firmly attached to the antibody via a chemical bond, and drug release depends on antibody degradation. This avoids non-specific linker cleavage in the bloodstream but has the drawback of a slower release mechanism, potentially affecting the effective intracellular drug concentration and thus efficacy [65]. They are more suitable for ADCs requiring stable, long-lasting release [65].

### 3.4. Mechanisms of Cytotoxic Payloads and Cell Death Pathways

#### 3.4.1. Classic Cytotoxic Mechanisms

The core of an ADC lies in the cytotoxic drug it carries, which exerts its killing effect on tumor cells through extremely potent cytotoxic actions. The cytotoxic drugs widely used in clinical and research settings currently fall into two main categories: microtubule inhibitors and DNA damaging agents. These two classes of drugs exert their cytotoxicity through distinct molecular mechanisms, representing the most classic and effective payloads in ADC design.

Microtubule inhibitors such as MMAE and DM1 (a maytansinoid) primarily interfere with microtubule assembly and dynamics, preventing spindle formation. This causes cell cycle arrest in metaphase, blocks cell division, and ultimately activates apoptotic pathways. Microtubules are tubular structures polymerized from α- and β-tubulin dimers, with an outer diameter of about 24 nm and an inner diameter of about 12 nm. They play crucial roles in cellular support and transport, are key components of the cytoskeleton, and are involved in chromosome segregation and the completion of mitosis. ADCs based on MMAE conjugated to antibodies like BV have demonstrated significant antitumor activity in various malignancies, with their targeted nature significantly reducing toxicity to normal cells [66] (Figure 3). Secondly, DNA damaging agents interfere with DNA replication and transcription by inducing DNA strand breaks or forming DNA adducts, thereby triggering apoptosis. For example, pyrrolobenzodiazepines (PBDs) can form interstrand cross-links with DNA, hindering DNA unwinding and replication, and possess extremely high cytotoxicity. Recent research indicates differences in DNA binding modes and toxicity profiles between mono-imine and bis-imine PBDs; mono-imine PBD conjugates demonstrate superior antitumor activity and lower renal toxicity both in vitro and in vivo, suggesting great potential as novel ADC payloads [67] (Figure 3).

#### 3.4.2. Novel Cytotoxic Mechanisms

In recent years, with continuous innovation in cell-killing technologies, various novel ADC payloads have emerged, expanding their mechanisms of action and applicability. Among these, spliceosome modulators and RNA polymerase inhibitors have become research hotspots, exhibiting unique antitumor killing modes by affecting gene expression and transcription processes in tumor cells.

Unlike traditional cytotoxic drugs, spliceosome modulators can regulate the splicing of pre-mRNA, leading to the accumulation of aberrant splicing events, thereby disrupting tumor cell survival and proliferation. RNA polymerase inhibitors, by blocking RNA synthesis, inhibit the expression of genes related to tumor cell growth, inducing cell cycle arrest and apoptosis. These mechanisms provide new targets and killing pathways for ADCs, potentially overcoming resistance to traditional cytotoxic drugs and improving antitumor efficacy. Furthermore, molecular glues and PROTAC conjugates, as emerging forms of protein degradation technology, also show great potential in the ADC field. Molecular glues induce the interaction between the target protein and the ubiquitin ligase complex, promoting ubiquitination and degradation of the target protein, achieving targeted clearance of pathogenic proteins. PROTAC conjugates work by recruiting E3 ubiquitin ligases to bind the target protein, inducing selective degradation of the target protein. Compared to the mechanism of traditional ADCs releasing cytotoxic drugs, protein degrader conjugates offer a targeted therapeutic strategy, enabling targeting of traditionally “undruggable” protein targets and broadening the application scope of ADCs. In summary, novel payloads such as spliceosome modulators, RNA polymerase inhibitors, molecular glue degraders, and PROTAC conjugates enrich the modes of action of ADCs through innovative molecular mechanisms, demonstrating unique tumor-killing capabilities [68,69] (Figure 2).

#### 3.4.3. Diversity of Cell Death Pathways

ADC-induced cell death pathways are diverse, primarily including apoptosis, pyroptosis, and necrosis, among others. These different death mechanisms profoundly impact therapeutic efficacy and potential side effects.

Firstly, apoptosis is the most common form of cell death induced by ADCs. Many ADC payloads, such as microtubule inhibitors or DNA damaging agents, can induce programmed apoptosis in tumor cells. Apoptosis, as a highly regulated programmed cell death, is typically accompanied by nuclear fragmentation and maintained membrane integrity, causing less inflammatory response and is thus considered a relatively gentle method of tumor cell clearance by ADCs. For example, the Nectin-4-MMAE ADC, after rapid internalization into cells, releases MMAE to initiate caspase-mediated apoptotic pathways, thereby inhibiting tumor cell growth [70]. Additionally, NOTCH3-ADC also achieves tumor regression by inducing apoptosis in receptor cells [71]. However, recent studies have found that ADCs can not only induce apoptosis but also activate other cell death pathways, particularly inflammatory forms of cell death like pyroptosis and necrosis.

Pyroptosis is a form of programmed necrosis accompanied by pore formation in the cell membrane and the release of inflammatory factors, characterized by membrane rupture mediated by the Gasdermin protein family. Research shows that ADCs containing Tubulysin payloads can induce Gasdermin protein sparking, triggering tumor cell pyroptosis, while also activating antitumor immune responses [72]. This mechanism enhances the activation of the tumor immune microenvironment, promoting dendritic cell maturation and infiltration of CD8+ cytotoxic T lymphocytes, which helps improve ADC therapeutic outcomes. However, the inflammatory nature of pyroptosis may increase the risk of treatment-related side effects, requiring a balance between the immune-activating effects and toxicity of the therapy. Necrosis, a passive, non-programmed form of cell death, is also accompanied by cell membrane rupture and release of cellular contents, triggering local inflammation. Certain novel ADCs, such as antibody-DNA nanostructure conjugates, can simultaneously activate caspase-3-mediated apoptosis and RIP3-mediated necroptosis-like death, enhancing tumor cell killing effects [73]. This dual-killing mechanism not only improves efficacy but was not associated with significant observed toxicity in the study.

Notably, the cell death mode induced by the payload has a critical impact on ADC efficacy and side effects. Apoptotic pathways tend to reduce inflammatory responses and are suitable for scenarios requiring gentle clearance of tumor cells. In contrast, pyroptosis and necrosis have immune-activating effects, can promote immune system involvement, and enhance antitumor responses, making them suitable for combination with immunotherapy strategies [74,75]. For example, when ADCs are combined with ICIs, ADC-induced immunogenic cell death (ICD) releases tumor neoantigens, activating the immune system and achieving synergistic therapeutic effects [75,76]. Furthermore, the nature of the payload and its release mechanism also directly influence the cell death pathway. For instance, microtubule inhibitor payloads like MMAE primarily induce apoptosis, whereas certain DNA damaging agents or RNA polymerase inhibitors may induce more complex forms of death [77,78].

### 3.5. Mechanisms of ADC Resistance

The resistance of ADC is not determined by a single mechanism, but rather is the cumulative result of multiple functional impairments occurring successively or simultaneously in various steps such as target antigen recognition, intracellular endocytosis and transport, effective payload release, and cytotoxic killing [79,80]. These mechanisms are interrelated and mutually influential, collectively forming a multi-dimensional barrier that limits the clinical efficacy of ADC. Firstly, the downregulation or complete loss of target antigen expression is the initial step of ADC failure, directly weakening the drug’s navigation ability [80]. Secondly, even if ADC successfully binds to the antigen, defects in its endocytosis, intracellular transport, and lysosomal degradation processes will also hinder the release of the payload drug. Finally, even if the effective payload drug is successfully released into the cytoplasm, tumor cells can still neutralize its cytotoxicity by activating survival signaling pathways or enhancing efflux mechanisms. Moreover, the tumor microenvironment (TME) also participates in the resistance process of ADC. The physical barriers of the TME (such as dense matrix) can hinder the penetration of ADC, and the immunosuppressive cells and cytokines within it can indirectly promote resistance by supporting tumor cell survival and reducing drug uptake [81,82]. In summary, the resistance of ADC is a multi-step, dynamic process, and these mechanisms do not act independently. They can coexist and interact in the same patient or the same tumor, greatly limiting the efficacy of ADC [81]. The following text will elaborate on the profound mechanisms in detail.

#### 3.5.1. Downregulation and Variation in Tumor Cell Antigen Expression

**Figure 4:**
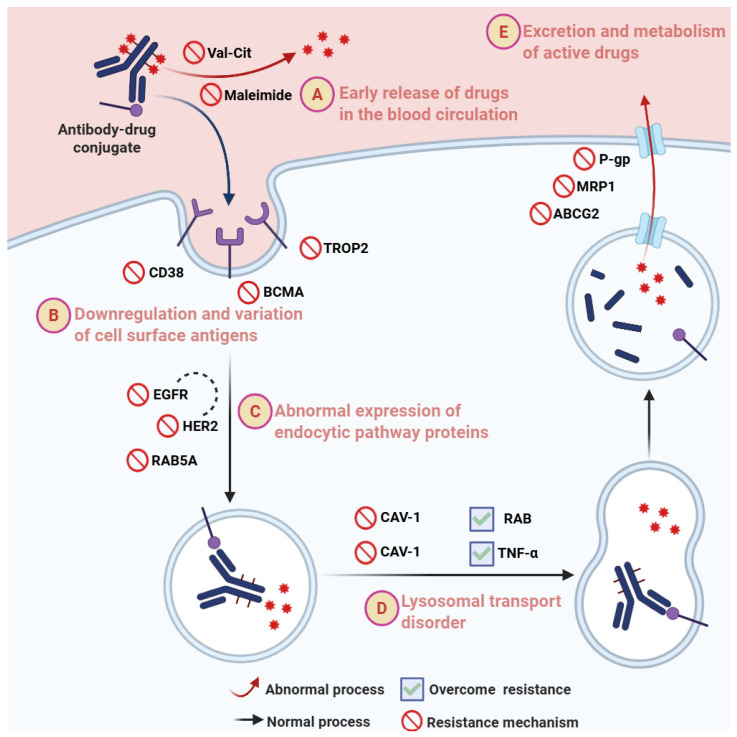
The mechanism of ADC resistance. In the circulation, Val-Cit dipeptide linkers and maleimide linkers are prone to reverse Michael addition reactions, leading to instability or even detachment of drug loading. After entering tumor cells, first, tumor cells evade the recognition and killing of ADCs by reducing the expression level of target antigens or undergoing antigen structural variations, which is an important mechanism of ADC treatment resistance. Commonly altered targets include CD38, BCMA, and TROP2. Secondly, abnormal expression of endocytic pathway proteins is also related to ADC treatment resistance, such as RAB5A and HER2, among which EGFR can form heterodimers with HER2, affecting the endocytic efficiency of ADCs. Additionally, the obstruction of endosome transport to lysosomes after endocytosis can also lead to ADC drug resistance, and this process is limited by the expression levels of CAV-1 and SLC46A3, while TNF-α and RAB family can reverse this phenomenon. Finally, the recognition and efflux by ABC transporters, especially the overexpression of P-gp, ABCG2, and MRP1, have become one of the important mechanisms of ADC resistance. ADC, antibody–drug conjugate; HER2, human epidermal growth factor receptor 2; CD38, Sialic acid-binding Ig-like lectin 38; BCMA, B cell maturation antigen; TROP2, Tumor-associated calcium signal transducer 2; CAV-1, Caveolin-1; SLC46A3, Solute Carrier Family 46 Member 3; P-gp, P-glycoprotein; ABCG2, ATP binding cassette subfamily G member 2 Gene; MRP1, Molecular factor-related protein 1. Created in BioRender. Liu, D. (2026) https://BioRender.com/tijh4mt (accessed on 9 February 2026).

Tumor cells evade ADC recognition and killing by reducing the expression level of the target antigen or undergoing antigen structural variations, which is an important mechanism leading to ADC treatment resistance. Specifically, tumor cells can reduce the number of cell surface antigens through various means such as gene expression regulation, accelerated protein degradation, and alternative splicing of the target, making it difficult for the ADC to bind effectively and release the drug intracellularly. For example, in multiple myeloma (MM), CD38 and BCMA are common targets; however, downregulation of BCMA on the surface of MM cells during treatment is one mechanism of drug resistance [30]. Additionally, structural variations or splicing of the tumor cell surface antigen can affect the binding affinity and internalization efficiency of the ADC. Taking biparatopic antibodies as an example, which recognize different non-overlapping epitopes on the same antigen, they can induce stronger target clustering and rapid internalization. This binding mode may help overcome resistance caused by antigen variation, but if key epitope mutations occur in the antigen, ADC binding capacity can still be limited [83]. In solid tumors with high heterogeneity, the spatial heterogeneity and temporal variation in antigen expression are more pronounced. For instance, TROP2, a target in various epithelial cancers, shows differential expression across different tumors and their metastases, and its expression can be downregulated in ADC-resistant cell lines, affecting ADC efficacy [84,85] (Figure 4).

The dynamic changes in antigen expression underscore the importance of dynamically monitoring the target antigen during ADC therapy. By detecting changes in antigen expression levels in real-time, clinicians can promptly adjust treatment strategies, such as switching targets, combining other immunotherapies, or using multi-specific ADCs, to delay or overcome the emergence of resistance. For example, in non-Hodgkin lymphoma, reduced CD20 expression leads to resistance to traditional anti-CD20 therapy. Research has found that complexes formed by CD20 and CD37 can stabilize CD20 targeting, suggesting the potential of selecting other targets with similar efficacy for ADC therapy when antigen expression decreases [86] (Figure 4).

#### 3.5.2. Alterations in Internalization and Trafficking Pathways

The efficacy of ADCs highly depends on their effective internalization and subsequent intracellular trafficking, particularly delivery to the lysosomes for drug release. However, reduced internalization efficiency and lysosomal dysfunction in tumor cells are important mechanisms leading to insufficient ADC efficacy and resistance.

Studies have indicated abnormal expression of endocytic pathway proteins in various drug-resistant tumor cells. For example, the expression of RAB5A, an early endosome marker, positively correlates with ADC sensitivity, and regulation of RAB family members can significantly affect ADC internalization and cytotoxic activity [87,88]. Another important example is HER2, which, as an ADC target, exhibits strong resistance to internalization. This resistance stems from the stability of HER2 and its recycling mechanism on the cell membrane [89]. Firstly, the ubiquitination level of HER2 directly affects its internalization efficiency; downregulated ubiquitination can inhibit the internalization of the ADC complex mediated by HER2, impairing ADC efficacy [90]. Secondly, abnormal EGFR expression and its heterodimer formation with HER2 can also influence ADC internalization efficiency [91]. Notably, lipid raft-mediated endocytosis also plays a key role in the cellular uptake of certain ADCs. Changes in lipid raft structure and the expression of related proteins like caveolin-1 can significantly affect ADC internalization efficiency and intracellular degradation, thereby impacting efficacy [50,92] (Figure 4).

Impaired trafficking of endosomes to lysosomes after internalization can also limit ADC drug release, promoting resistance [49]. For example, T-DM1 primarily enters HER2-positive cancer cells via lipid raft-mediated endocytosis, and this process depends on caveolin-1 (CAV-1) expression levels. Overexpression of CAV-1 significantly promotes T-DM1 internalization and lysosomal degradation, whereas disruption of lipid raft structure and excessive activation of HER2 inhibit this process [50]. Furthermore, SLC46A3, a lysosomal membrane protein, is involved in the transport of the main metabolites of T-DM1, and its functional defects can also lead to insufficient drug release [93,94] (Figure 4).

Regulatory molecules involved in internalization and trafficking pathways have become potential targets for reversing ADC resistance. For instance, Hsp90 inhibitors can induce HER2 degradation, enhancing ADC cellular uptake and antitumor effects [89]. Enhancing lipid raft-mediated endocytosis or promoting non-canonical EGFR internalization can also improve ADC efficacy. p38-mediated, ligand-independent EGFR internalization was found to promote intracellular delivery of ADCs; in this process, tumor necrosis factor-α (TNF-α) can induce rapid internalization of the cetuximab-EGFR complex via p38 phosphorylation of EGFR, reactivating intracellular delivery in ADC-resistant settings and enhancing antitumor effects [51]. Meanwhile, genetic or pharmacological intervention targeting key endocytic regulatory proteins like RAB5 and RAB4a also provides strategies for improving ADC internalization and drug release [87,88]. Finally, functional imaging technologies based on endocytic mechanisms have been developed for real-time monitoring of ADC cellular uptake and metabolic processes, providing technical support for in-depth analysis of resistance mechanisms and optimization of ADC design [92].

#### 3.5.3. Linker Stability Dysregulation and Structural Heterogeneity

The stability of the linker in ADCs directly impacts therapeutic efficacy and safety. If the linker cleaves prematurely in the bloodstream, leading to non-specific release of the payload, it not only reduces the efficiency of targeted tumor delivery but can also cause severe systemic toxicity. Existing research points out that various peptidases in the blood can cleave certain peptide-based linkers. For example, the widely used valine-citrulline (Val-Cit) dipeptide linker, while effective for intracellular cleavage, carries the risk of premature cleavage in the circulation by non-target proteases such as elastase, leading to premature drug release and toxicities like bone marrow suppression [95]. Additionally, maleimide-based linkers, which form thiosuccinimide rings upon conjugation to antibody cysteines, are also prone to retro-Michael reactions in the blood, leading to instability or even detachment of the drug payload [96]. To address these issues, researchers have employed various strategies to optimize linker design and improve its stability in plasma. For example, introducing hydrophilic glutamate residues at specific positions can reduce ADC aggregation induced by hydrophobic payloads, improve the DAR, and significantly decrease non-specific release [97]. Simultaneously, tandem cleavage linker strategies, incorporating two enzyme-protective groups within the linker, enhance stability in circulation while enabling effective payload release in target cells, significantly improving the therapeutic index of ADCs [95] (Figure 4).

Structural heterogeneity in ADCs primarily stems from the non-uniform conjugation of linkers to antibodies, including different conjugation sites and varying DAR values. Heterogeneity not only affects the physical stability of ADCs but also leads to variations in drug release efficiency and pharmacokinetics [97,98]. To address this issue, various site-specific conjugation technologies have been developed in recent years, such as THIOMAB and AJICAP technologies. By precisely controlling the conjugation site, they enable the production of homogeneous ADCs, significantly improving stability and efficacy [42,98]. For instance, incorporating hydrophilic polyethylene glycol (PEG) units into the linker design can effectively shield hydrophobic payloads, reduce ADC aggregation tendency, and improve pharmacokinetic properties [99,100]. Furthermore, studies indicate that neighboring residues in the linker microenvironment significantly influence the hydrolytic stability of the linker. Therefore, rational design of the spatial structure between the linker and the antibody can promote stable hydrolysis of the linker and enhance the overall stability of the ADC [61].

#### 3.5.4. Drug Efflux and Metabolism

In addition to the aforementioned mechanisms involving tumor cell surface antigen inactivation, altered internalization/trafficking pathways, and linker dysfunction, the efflux and metabolism of the active cytotoxic drug are also key factors leading to reduced ADC efficacy.

Drug efflux pumps, particularly P-glycoprotein (P-gp) from the ATP-binding cassette (ABC) transporter family, play a significant role in ADC resistance. Studies show that many commonly used ADC cytotoxic drugs, such as calicheamicin gamma1, monomethyl auristatin E (MMAE), DM1, and DM4, are substrates of P-gp and are easily actively effluxed by P-gp, leading to reduced intracellular effective drug concentration and consequently weakening the cytotoxic effect of the ADC [101] (Figure 4). Additionally, pyrimidine-based drugs like SJG136 and SGD-1882 are also recognized and effluxed by various ABC transporters including P-gp, ABCG2, and MRP1 [101]. Therefore, upregulation of drug efflux pump expression, especially P-gp, by tumor cells is an important mechanism of ADC resistance [102]. To counter this mechanism, current research proposes using novel ADC payloads that are less recognized by ABC transporters, or combining ADCs with small molecule inhibitors, siRNA, etc., to downregulate efflux pump expression and enhance intracellular accumulation and killing activity of ADCs [103,104]. Furthermore, changes in the activity of drug-metabolizing enzymes also impact the concentration of the active drug from ADCs. The payload of an ADC needs to be cleaved and released or modified by intracellular enzyme systems for the active drug to exert its cytotoxic effect. However, variations in metabolic enzyme activity can lead to excessively rapid or slow metabolism of the active drug, affecting its intracellular concentration and efficacy. For example, the calicheamicin-based payload in ABBV-011 is released through enzymatic dissociation in vivo, but hydrolysis of a key disulfide bond by hydrolases is a potential critical defect that may affect the overall stability of the ADC [105].

## 4. Clinical Applications of ADCs in Malignant Tumors

ADCs have revolutionized the treatment landscape for malignant tumors through their “targeted delivery and killing” design concept. The synergistic combination of the targeting antibody, linker, and cytotoxic payload has driven the advancement of ADC technology [106]. Currently, the FDA has approved several ADC drugs for the treatment of hematological malignancies such as lymphoma and leukemia, as well as various solid tumors including breast cancer, gastric cancer, and urothelial carcinoma.

### 4.1. Application of ADCs in Hematological Malignancies

#### 4.1.1. Acute Myeloid Leukemia (AML)

The treatment of AML long relied on chemotherapy with poor prognosis, particularly for patients with relapsed/refractory (R/R) disease or those with specific genetic risks. The emergence of ADCs, especially those targeting CD33, a myeloid cell surface antigen expressed on over 90% of AML blasts, has made it an ideal therapeutic target [107,108].

Gemtuzumab Ozogamicin (GO), the first ADC approved by the FDA, targets the CD33 antigen expressed on more than 90% of AML blasts and is conjugated via a cleavable linker to the potent DNA-damaging agent calicheamicin [109,110]. Its mechanism of action involves GO binding to CD33, followed by receptor-mediated endocytosis. Within the acidic environment of the lysosome, the linker is hydrolyzed, releasing calicheamicin molecules that translocate to the nucleus, bind to the DNA minor groove, cause double-strand breaks, and ultimately lead to apoptosis [109].

The initial clinical application of GO was based on a Phase II study (142 patients with CD33-positive AML in first relapse, Objective Response Rate [ORR] of 30%), leading to its approval in 2000 [15]. However, subsequent Phase III studies found that adding a higher dose of GO (9 mg/m^2^) to standard induction chemotherapy failed to improve complete response rates, relapse-free survival, or overall survival (OS) in younger AML patients, while increasing induction mortality and the risk of hepatic veno-occlusive disease (VOD), leading to its voluntary withdrawal from the market in 2010 [111]. This setback prompted re-evaluation of GO’s dosing regimen and beneficiary population. A series of subsequent key studies, such as the ALFA-0701 Phase III trial, demonstrated that fractionated lower doses (3 mg/m^2^ on days 1, 4, and 7) of GO combined with chemotherapy significantly improved event-free survival, reducing the risk by 34%, and showed a trend towards OS benefit compared to chemotherapy alone in newly diagnosed CD33-positive AML patients [112]. Based on these rigorous dose optimization data, GO was re-approved in 2017, establishing its role as a standard treatment for newly diagnosed CD33-positive AML, particularly in patients with intermediate or low-risk features [109]. Furthermore, studies have found that the efficacy of GO is influenced by factors such as CD33 expression levels, CD33 single nucleotide polymorphisms, and baseline liver function, while myelosuppression and VOD remain its major toxicities requiring close monitoring and management [15].

#### 4.1.2. Acute Lymphoblastic Leukemia

In the field of ALL, ADCs have also achieved breakthrough progress, providing effective salvage therapy and a bridge to transplantation for R/R patients, changing the previously limited and ineffective treatment options. Inotuzumab Ozogamicin (InO) targets CD22 on B cells and is also armed with calicheamicin as its cytotoxic payload [110]. Unlike GO, CD22 rapidly recycles to the cell surface after antibody–CD22 complex internalization, facilitating the entry of more ADC molecules into the cell and enhancing the killing effect. The Phase III INO-VATE study confirmed that compared to standard chemotherapy, InO significantly increased the CR rate (73.8% vs. 30.9%) and the rate of CR with incomplete hematologic recovery (CRi), prolonged median OS (7.7 months vs. 6.2 months), and enabled more patients to successfully bridge to hematopoietic stem cell transplantation (HSCT) (48.2% vs. 22.2%) in R/R B-cell acute lymphoblastic leukemia (B-ALL) patients, offering the potential for long-term benefit [113]. Long-term follow-up data further consolidated its survival benefit and confirmed post-transplant survival advantage [113]. Beyond later-line therapy, InO has shown potential in the frontline setting. A study exploring InO combined with dexamethasone as first-line induction therapy for older patients (>55 years) with Philadelphia chromosome-negative B-ALL showed that all evaluable patients achieved CR, and 71% achieved molecular remission after induction, with a 3-year OS rate as high as 73%, providing an effective and relatively safe new option for this less tolerant population [114]. However, the primary risk associated with InO is VOD, particularly increased in patients undergoing HSCT or with a history of liver disease, necessitating strict liver function monitoring, fractionated dosing strategies, and adequate washout periods before and after transplantation [113,115]. The risk of VOD associated with InO, particularly when sequenced with HSCT [113], underscores the critical importance of linker stability, as premature release of the calicheamicin payload is considered a key contributor to this toxicity [110]. Consequently, the clinical experience with InO has strongly motivated the pursuit of circulatory stability across the ADC field, favoring the design of plasma-inert linkers in subsequent candidates. It also established clinical management norms—including fractionated dosing and adequate washout periods before and after transplantation—that have become standard risk mitigation strategies for high-risk ADC development. For some targets, the kinetics of internalization may hold greater prognostic value than membrane expression levels alone. By transforming refractory ALL from a palliative condition to a treatable state with the potential for long-term survival via bridging to transplant, InO highlights the unique value of ADCs as potent remission inducers rather than mere substitutes for traditional chemotherapy.

#### 4.1.3. Lymphoma

Lymphoma is one of the most active and fruitful areas for ADC development, with multiple ADCs targeting different antigens bringing transformative treatments to patients with various lymphomas. Their application has progressively moved from salvage therapy to frontline and even consolidation settings. BV targets CD30, an antigen highly expressed in classical Hodgkin lymphoma (cHL) and systemic anaplastic large cell lymphoma (sALCL) [116]. BV consists of an anti-CD30 antibody conjugated via a cathepsin B-cleavable linker to the microtubule inhibitor monomethyl auristatin E (MMAE) [117]. Its efficacy stems from multiple mechanisms: first, upon binding CD30, it is efficiently internalized, and MMAE is released in the lysosome, inducing G2/M phase cell cycle arrest and apoptosis by inhibiting tubulin polymerization. Second, due to its hydrophobicity, free MMAE can penetrate cell membranes and exert a “bystander effect” on neighboring CD30-negative tumor cells or supportive cells in the tumor microenvironment, which is crucial in heterogeneous tumor environments [118]. Furthermore, recent research has found that BV-driven microtubule disruption can trigger endoplasmic reticulum stress, leading to calreticulin exposure, and release of ATP and high mobility group box 1 (HMGB1), thereby inducing immunogenic cell death, which further activates dendritic cells and cytotoxic T cells, providing a solid theoretical basis for combining ADCs with ICIs [119].

In the pivotal Phase II trial for R/R cHL after autologous hematopoietic stem cell transplantation (ASCT), BV monotherapy achieved an ORR of 75% and a CR rate of 34%, with a median progression-free survival (PFS) of 5.6 months, and a median duration of response (DOR) of 20.5 months for complete responders [120]. Based on this, its indication was expanded to consolidation therapy. The AETHERA Phase III study showed that for cHL patients at high risk of relapse post-ASCT, BV consolidation therapy significantly prolonged median PFS (42.9 months vs. 24.1 months), establishing its role in preventing relapse after transplantation [121]. The ECHELON-1 study successfully moved it to the frontline, demonstrating that the A + AVD regimen significantly improved the 3-year PFS rate compared to the ABVD regimen (83.1% vs. 76.0%) in stage III/IV cHL patients, while avoiding the pulmonary toxicity associated with bleomycin [122]. Recently, BV has even shown potential across indications in diffuse large B-cell lymphoma (DLBCL). The ECHELON-3 trial indicated that BV combined with lenalidomide and rituximab significantly improved median OS (13.8 months vs. 8.5 months) and median PFS (4.2 months vs. 2.6 months) compared to the control group in heavily pretreated R/R DLBCL patients, with benefit independent of CD30 expression level, offering new hope for patients who have failed multiple lines of therapy [123]. The main adverse effects of BV include peripheral neuropathy and neutropenia, which are mostly manageable, and peripheral neuropathy often improves after discontinuation or symptomatic treatment [120,122]. The dose-limiting toxicities of BV (peripheral neuropathy and myelosuppression) represent the intrinsic toxicity profile of its microtubule inhibitor payload, MMAE [117]. This clinical observation confirms that even with targeted delivery, the class-specific toxicity of the payload remains a decisive factor for ADC safety. This has incentivized the exploration of novel payloads with distinct mechanisms of action and toxicity profiles, aiming to provide patients with therapeutic options offering comparable efficacy but differentiated side-effect spectra.

Polatuzumab Vedotin (Pola) targets CD79b, a key component of the B-cell receptor, and is the first ADC to show significant survival benefit in R/R DLBCL, breaking the long-standing lack of effective treatments in this setting [124]. The POLARIX Phase III study brought Pola to the frontline, demonstrating that the Pola plus rituximab, cyclophosphamide, doxorubicin, and prednisone (Pola-R-CHP) regimen significantly improved the 2-year PFS rate compared to the standard rituximab, cyclophosphamide, doxorubicin, vincristine, and prednisone (R-CHOP) regimen (76.7% vs. 70.2%) in previously untreated intermediate-high risk DLBCL patients, reducing the risk of disease progression, relapse, or death by 27%. This provides a new standard treatment option for such patients, representing the first major breakthrough in frontline DLBCL therapy in 20 years [125]. Combination strategies for Pola are also being explored. A Phase Ib/II study showed that Pola combined with the CD20xCD3 bispecific antibody Mosunetuzumab achieved an ORR of 59.2% and a CR rate of 45.9%, with a median PFS of 11.4 months and manageable safety in transplant-ineligible R/R LBCL patients, demonstrating the great potential of synergistic effects between ADCs and immunotherapies [126].

CD19 is a key molecule for B-cell development, activation, and signaling, stably and highly expressed in the vast majority of B-cell malignancies, making it another critical target [127]. Loncastuximab Tesirine (Lonca) is an ADC targeting CD19 and carrying a PBD dimer payload. PBD is a potent DNA cross-linking agent causing irreversible DNA damage, differing from the mechanism of MMAE, and is effective against cells in different cell cycle stages [128]. In a Phase II study, Lonca achieved an ORR of 48.3% and a CR rate of 24.1% in heavily pretreated R/R DLBCL patients (median prior lines of therapy was 3), with a median DOR of 10.3 months, demonstrating significant activity in this highly refractory population [129]. Additionally, Lonca combined with rituximab showed promise in R/R follicular lymphoma, with a CR rate as high as 67% at 12 weeks, offering new therapeutic hope for patients with this typically incurable indolent lymphoma [130].

In preclinical studies, novel ADCs continue to emerge, showing better therapeutic potential and safety. For example, ADCT-602, targeting CD22, employs a humanized antibody site-specifically conjugated to the PBD dimer tesirine, ensuring a homogeneous drug-to-antibody ratio (DAR). It demonstrated potent, dose-dependent antitumor activity in CD22-positive lymphoma and leukemia models, and due to linker optimization, exhibited favorable stability and tolerability [131]. Another ADC, BAY-943, targeting the interleukin-3 receptor alpha chain (IL3RA or CD123), combines a high-affinity anti-IL3RA antibody with a novel kinesin spindle protein inhibitor payload. It showed significant antitumor effects in preclinical models of acute myeloid leukemia and Hodgkin lymphoma. Given CD123’s high expression on leukemia stem cells, it may potentially target tumor-initiating cells, while preclinical data indicated a good safety window [132].

For Hairy Cell Leukemia (HCL), a rare B-cell neoplasm, the anti-CD22 immunotoxin Moxetumomab Pasudotox has made significant progress. A pivotal Phase III study showed that this drug achieved a durable CR rate of 30% and an ORR of 75% in patients with R/R HCL after purine analog therapy. Most patients achieving CR also achieved minimal residual disease (MRD) negativity by immunohistochemistry, suggesting potential long-term disease control or even potential cure, offering unprecedented deep response opportunities for these refractory patients [133]. In the field of lymphoma, we clearly demonstrate the complete evolution of ADCs from later-line to frontline therapy and from monotherapy to combination regimens, revealing the appropriate contexts for different design strategies. The success of BV (based on MMAE) validates the efficacy of the protease-cleavable linker plus potent membrane-permeable microtubule inhibitor strategy in CD30-positive lymphomas, where its bystander effect is crucial for addressing the tumor microenvironment in Hodgkin lymphoma. The breakthrough of Pola (targeting CD79b) in DLBCL indicates that targeting a core component of the B-cell receptor enables efficient tumor-specific delivery, even when the target antigen expression level is not exceptionally high. Lonca (based on a PBD dimer) demonstrates that employing a payload with a different mechanism of action can overcome cross-resistance in highly refractory patients. Collectively, these experiences underscore that in hematological malignancies, lineage specificity of the target and a reliable internalization pathway are the cornerstones of ADC success, while the choice of the linker-payload system dictates the depth of efficacy and potential for combination therapy.

#### 4.1.4. Multiple Myeloma (MM)

Although multiple treatment options exist for multiple myeloma, the prognosis remains very poor for triple-class exposed/refractory (TCE/TCR) patients, necessitating novel mechanism drugs [134]. Belantamab Mafodotin (Belamaf) is the first ADC targeting BCMA, which is universally highly expressed on plasma cells and MM cells and is a key factor for their survival and proliferation. Belamaf is conjugated via a non-cleavable linker to the microtubule inhibitor monomethyl auristatin F (MMAF). After internalization, it is fully degraded within the lysosome, releasing a charged MMAF derivative. Due to its charge, it has limited ability to penetrate cell membranes, thus restricting the “bystander effect,” but this may also help reduce toxicity to surrounding normal tissues [135]. Its approval was based on the DREAMM-2 Phase II study, which showed an ORR of approximately 31% in triple-refractory MM patients. However, in the subsequent head-to-head DREAMM-3 Phase III study comparing Belamaf monotherapy to pomalidomide plus dexamethasone (Pd), Belamaf monotherapy did not significantly improve PFS (median PFS 11.2 months vs. 7.0 months), suggesting its advantage as a later-line monotherapy may be limited, also reflecting the high challenges of later-line MM treatment [136]. This result has shifted the research focus towards combination therapy. A subgroup analysis from another trial showed that Belamaf combined with the Pd regimen (Belamaf-Pd) achieved a remarkably high ORR of 86.4% and a median PFS of 18.3 months in TCE/TCR R/R MM patients, providing a new option with durable responses for this extremely refractory patient group [137]. Belamaf’s unique and common adverse effect is corneal epithelial changes, which can lead to visual impairment and photophobia. The mechanism may be related to non-specific uptake of MMAF in BCMA-expressing corneal epithelial cells. Therefore, regular ophthalmological monitoring and individualized dose adjustment strategies are mandatory during treatment [136,137]. This unique corneal toxicity serves as a classic case of “on-target, off-tumor” toxicity. The combination of low-level BCMA expression on corneal epithelium and the intracellular accumulation of the charged, membrane-impermeable MMAF metabolite released via its non-cleavable linker collectively underlies this adverse reaction [135]. This outcome has profoundly influenced subsequent therapeutic strategies targeting BCMA and highlights a critical consideration in ADC design: the need to evaluate the retention potential of the payload and its metabolites in normal tissues, based on their physicochemical properties, and the consequent risk of organ-specific toxicity.

While Belamaf’s design restricts the bystander effect and reduces off-target toxicity, it may also limit its diffusible killing capacity within tumor tissues. The failure of the DREAMM-3 study to meet its primary endpoint suggests that for a disease like MM, characterized by a highly heterogeneous and immunosuppressive microenvironment, the simple paradigm of “targeted delivery + potent killing” may be insufficient as a later-line monotherapy. Subsequent combination therapies (e.g., Belamaf-Pd) demonstrated significantly higher response rates, strongly indicating that in complex diseases like myeloma, the future of ADCs leans more towards serving as backbone agents for immunomodulatory or combination regimens. Its unique corneal toxicity also serves as a warning that low-level expression of the target antigen in critical normal tissues can, via the active uptake mechanism of ADCs, precipitate organ-specific toxicity.

### 4.2. Application of ADCs in Solid Malignant Tumors

In the field of solid tumor therapy, ADC drugs targeting human epidermal growth factor receptor 2 (HER2), Trophoblast Cell Surface Antigen 2 (TROP2), Nectin-4, TF, Folate Receptor Alpha (FRα), among others, have achieved remarkable success, profoundly changing the treatment landscape for various malignancies including breast cancer, gastric cancer, urothelial carcinoma, gynecological cancers, and lung cancer [138,139]. The following sections detail the clinical progress of ADCs by tumor type.

#### 4.2.1. Breast Cancer

Breast cancer is the most mature field for ADC development and application, achieving milestone successes, particularly in the HER2-positive subtype. HER2-positive breast cancer accounts for about 15–20% of all breast cancers and is associated with strong invasiveness and poor prognosis. Furthermore, ADCs targeting other targets like TROP2 have expanded treatment options for hormone receptor-positive/HER2-negative (HR+/HER2-) and triple-negative breast cancer (TNBC) [140].

The first-generation HER2-targeting ADC T-DM1 (trastuzumab emtansine) combines the targeting ability of trastuzumab with the microtubule inhibitor DM1 via a stable non-cleavable linker. Its approval was based on the EMILIA Phase III trial, which established T-DM1’s role as a second-line treatment for HER2-positive metastatic breast cancer patients previously treated with trastuzumab and a taxane. Compared to lapatinib plus capecitabine, T-DM1 significantly improved PFS (9.6 months vs. 6.4 months) and OS (30.9 months vs. 25.1 months), with a better safety profile [17]. In early breast cancer, for patients who did not achieve pathological complete response after neoadjuvant therapy, adjuvant T-DM1 treatment post-surgery significantly reduced the risk of recurrence or death by 50% compared to trastuzumab monotherapy, establishing its role in the adjuvant setting [7]. The improved safety profile of T-DM1 compared to traditional chemotherapy (aside from thrombocytopenia) validates the strategic advantage of its non-cleavable linker (MCC thioether), which requires complete lysosomal degradation of the antibody to release the active metabolite, thereby effectively limiting systemic free DM1 exposure [17]. Concurrently, its efficacy being strictly confined to the HER2-high population clearly defines the design boundaries of first-generation ADCs.

However, tumor heterogeneity and resistance mechanisms prompted the development of next-generation ADCs. Trastuzumab Deruxtecan (T-DXd, DS-8201), as a second-generation HER2-targeting ADC, has achieved greater therapeutic benefits. First, it uses a humanized anti-HER2 antibody for precise targeting. Second, its cleavable tetrapeptide linker is efficiently cleaved by lysosomal proteases highly expressed in tumor cells to release the payload. Moreover, its payload, the topoisomerase I inhibitor DXd, has strong cell membrane permeability. Finally, its high DAR of 8 ensures sufficient cytotoxic drug is delivered to tumor cells [141]. These properties collectively confer upon T-DXd a powerful “bystander effect”—after killing HER2-positive cells, the released DXd can diffuse and kill adjacent HER2-low or negative tumor cells, which is crucial for overcoming tumor heterogeneity [142].

A Phase III trial directly compared T-DXd with T-DM1 in the second-line treatment of HER2-positive metastatic breast cancer. The results were impressive: the 12-month PFS rate was 75.8% in the T-DXd group, far superior to 34.1% in the T-DM1 group, with a 72% reduction in the risk of disease progression or death (Hazard Ratio [HR] = 0.28). Concurrently, the ORR in the T-DXd group was nearly 80%, significantly higher than in the T-DM1 group [143]. This study undoubtedly established T-DXd as the new standard for second-line therapy in HER2-positive advanced breast cancer. Furthermore, the success of T-DXd has redefined the boundaries of HER2-targeted therapy, creating a new therapeutic strategy for “HER2-low” expression. HER2-low (defined as immunohistochemistry [IHC] 1+ or IHC 2+/in situ hybridization [ISH] negative) accounts for about 45–55% of all breast cancers and was previously considered HER2-negative, unable to benefit from traditional HER2-targeted therapy. The DESTINY-Breast04 study first confirmed that in pretreated patients with HER2-low metastatic breast cancer (including both HR+ and TNBC), T-DXd compared to physician’s choice of chemotherapy significantly prolonged both PFS and OS [8]. This discovery established HER2-low as a new targetable biomarker.

Recently reported studies have further extended the benefit to patients with endocrine therapy-resistant, HR-positive, HER2-low, and even “ultra-low” expressing metastatic breast cancer. Results showed that compared to standard chemotherapy, T-DXd significantly extended median PFS from 8.1 months to 13.2 months, reducing the risk of disease progression or death by 38% [144]. This indicates that T-DXd’s activity is continuous across the HER2 expression spectrum, and its potential patient population may be broader than initially thought. The fundamental reason for the differential efficacy of T-DM1 and T-DXd across the HER2 expression spectrum lies in their distinct pharmacologic behaviors dictated by core design parameters, particularly the membrane permeability of the payload and the resulting bystander effect. T-DM1 employs a non-cleavable linker, and its released charged metabolite (Lys-MCC-DM1) has poor cell membrane permeability. Its killing effect is strictly confined to HER2-high cells that have successfully internalized the ADC, with no significant impact on adjacent low-expressing or negative cells [145]. In contrast, T-DXd features a higher DAR, utilizes a cleavable linker, and carries the highly membrane-permeable topoisomerase I inhibitor, DXd. Upon release within target cells, DXd freely diffuses into the surrounding microenvironment, killing neighboring tumor cells with low antigen expression, thereby overcoming HER2 heterogeneity [142]. Consequently, the “diffusibility” of the payload and the strength of the bystander effect are critical design elements determining whether an ADC can break the dependence on traditional high-expression targets and expand the treatable patient population. Of course, the widespread application of T-DXd comes with specific safety considerations. Across multiple studies, the incidence of interstitial lung disease/pneumonitis (ILD) ranges from 10 to 15%, making it the most notable adverse reaction. Although most cases are mild to moderate, fatal events have been reported, necessitating vigilant monitoring and prompt management in clinical practice [143,146]. T-DXd-associated interstitial lung disease (ILD) is a toxicity warranting vigilance. This co-existence of the toxicity and efficacy profiles perfectly illustrates the design trade-off in ADCs: maximizing the “bystander effect” to overcome heterogeneity may simultaneously increase the risk of injury to specific organs. This has directly driven the implementation of more stringent and prospective monitoring and management for ILD in the clinical development of subsequent ADCs (e.g., Dato-DXd) and motivates the exploration of novel ADC designs aimed at decoupling potent anti-tumor activity from more controllable pulmonary toxicity.

Besides HER2, TROP2 is another important ADC target in breast cancer. TROP2 is highly expressed in most TNBC and HR+/HER2- breast cancers and is associated with tumor proliferation, invasion, and poor prognosis. Sacituzumab Govitecan (SG) is the first approved TROP2-targeting ADC, consisting of a humanized anti-TROP2 antibody, a hydrolysable linker, and the topoisomerase I inhibitor SN-38 (the active metabolite of irinotecan). Its approval was based on the ASCENT Phase III study, which demonstrated in pretreated metastatic TNBC patients that SG compared to single-agent chemotherapy significantly improved median PFS (5.6 months vs. 1.7 months) and median OS (12.1 months vs. 6.7 months) [147]. Subsequently, the TROPiCS-02 Phase III study conducted in HR+/HER2- advanced breast cancer also met its primary endpoint, with final analysis showing that SG significantly improved median OS compared to chemotherapy (14.4 months vs. 11.2 months), providing a new later-line option for these endocrine-resistant patients [148]. A Phase IIb trial in a Chinese population further validated the efficacy and manageable safety of SG, with an ORR reaching 38.8% [149].

Another notable TROP2-targeting ADC is datopotamab deruxtecan (Dato-DXd), which also uses DXd as its cytotoxic payload. The Phase III TROPION-Breast01 study showed that in previously treated inoperable or metastatic HR+/HER2- breast cancer, Dato-DXd compared to investigator’s choice of chemotherapy significantly prolonged PFS (median PFS: 6.9 months vs. 4.9 months). More importantly, Dato-DXd exhibited a superior safety profile, with a significantly lower incidence of grade 3 or higher treatment-related adverse events than the chemotherapy group (21% vs. 45%) [150]. Additionally, in a Phase I study, Dato-DXd showed promising antitumor activity in advanced TNBC, supporting its further exploration in this area [151].

Beyond HER2 and TROP2, ADCs targeting other antigens are under active development to address unmet clinical needs. For example, B7-H4 (CD276) is an immunomodulatory protein overexpressed in various solid tumors (including breast and ovarian cancer), emerging as a new target with high therapeutic value. B7-H4-based ADC drugs like YL201 and XMT-1660 have shown potent antitumor activity in preclinical models. XMT-1660 utilizes an optimized Dolasynthen (DS) platform for precise DAR control and site-specific conjugation and has now entered Phase I clinical trials (NCT05377996), offering a new treatment option for patients with B7-H4-positive tumors [152,153]. Furthermore, ADCs targeting HER3, Frizzled-7 (FZD7), among others, are also undergoing early clinical evaluation in breast cancer, further enriching the treatment arsenal [154,155].

Breast cancer provides the most vivid illustration of ADC design evolution and its clinical impact. T-DM1, with its non-cleavable linker ensuring high plasma stability, exhibits efficacy strictly dependent on HER2-high expression and efficient internalization. In contrast, T-DXd achieves a paradigm shift through its cleavable linker, high DAR, and highly membrane-permeable DXd payload. It transforms ADCs from therapies targeting driver oncogenes into targeted chemotherapy directed at tumor-associated antigens. Its efficacy stems not only from killing HER2-high cells but, more importantly, from overcoming intratumoral heterogeneity via a potent bystander effect, thereby opening the entirely new therapeutic domain of HER2-low breast cancer. This demonstrates that in solid tumors, the diffusibility of the payload may be more determinative of therapeutic potential than the antibody’s binding affinity. In comparison, the exploration of SG and Dato-DXd targeting TROP2 validates the feasibility of selecting highly expressed, yet non-driver, antigens in HR+/HER2- breast cancer. The key lies in the payloads (SN-38 or DXd) themselves possessing sufficient cytotoxicity and the linkers enabling efficient release. However, the risk of T-DXd-associated ILD sharply raises a new question: while pursuing broader efficacy and stronger bystander effects, how do we predict and manage the novel organ-specific toxicities that arise? This may become the core dilemma that next-generation ADC design must confront.

#### 4.2.2. Gastric Cancer and Gastroesophageal Junction ADENOCARCINOMA

Gastric cancer is a common malignancy worldwide, with HER2 overexpression or amplification occurring in approximately 7.3–20.2% of advanced cases [156]. Although trastuzumab combined with chemotherapy is the first-line standard, later-line options are limited, and resistance often develops. ADC drugs offer new hope for HER2-positive gastric cancer patients, while ADCs targeting new targets like Claudin18.2 (CLDN18.2) also show great potential. The success of T-DXd in gastric cancer is remarkable. A Phase II study compared T-DXd versus investigator’s choice chemotherapy in patients with previously trastuzumab-treated HER2-positive advanced gastric/gastroesophageal junction adenocarcinoma. Results showed that T-DXd achieved significant improvements in both ORR (51% vs. 14%) and OS (median OS: 12.5 months vs. 8.9 months) [157]. These results led to the approval of T-DXd for later-line treatment of HER2-positive gastric cancer in many countries worldwide. While T-DXd has proven effective in HER2-positive gastric cancer, its efficacy is minimal in HER2-low gastric cancer, a stark contrast to its performance in breast cancer. This suggests that HER2-low may not be a universal predictive biomarker; its therapeutic value likely depends on the inherent sensitivity of the tumor tissue to the specific payload (e.g., DXd).

Notably, although the DESTINY-CRC02 study primarily focused on colorectal cancer, it compared two doses of T-DXd (5.4 mg/kg vs. 6.4 mg/kg) for efficacy and safety, finding that the 5.4 mg/kg dose group not only had considerable efficacy but also better safety, with lower incidence and severity of ILD compared to the 6.4 mg/kg group [158]. This finding provides important reference for optimizing T-DXd dosing regimens and balancing efficacy and risk in gastrointestinal tumors, including gastric cancer.

The novel HER2-targeting ADC RC48 (Disitamab Vedotin), which uses MMAE as its cytotoxic payload, has also made significant progress in gastric cancer. A single-arm Phase II study involving 125 patients with HER2-overexpressing (IHC 2+/3+) locally advanced or metastatic gastric/gastroesophageal junction cancer showed that after multiple lines of therapy, patients treated with RC48 monotherapy achieved an ORR of 24.8%, median PFS of 4.1 months, and median OS of 7.9 months, demonstrating promising application prospects [159]. RC48 (Disitamab Vedotin), which utilizes the MMAE payload, demonstrates the feasibility of an alternative design in gastric cancer. However, its distinct efficacy profile compared to T-DXd provides a rationale for subsequent sequential or combination strategies. Claudin-18.2 (CLDN18.2) is a stomach-specific tight junction protein abnormally expressed in approximately 30–60% of gastric adenocarcinomas, making it a highly potential new target. CMG901 is a CLDN18.2-targeting ADC that has entered Phase III clinical trials. Preclinical and early clinical data indicate that CMG901 exhibits potent antitumor activity and manageable toxicity in CLDN18.2-positive gastric cancer models [160]. The Phase III SPOTLIGHT trial confirmed the survival benefit of the CLDN18.2-targeting monoclonal antibody Zolbetuximab combined with chemotherapy in the first-line setting, which undoubtedly provides strong support for CLDN18.2-targeted therapies (including ADCs) in this field [161]. The emergence of CLDN18.2 as a stomach-specific target marks the entry of ADC development into a new phase focused on “tissue-restricted antigens,” aiming to maximize the therapeutic window. Collectively, these advances demonstrate that in cancers with strong heterogeneity and limited treatment options, such as gastric cancer, ADC development necessitates more refined patient stratification and biomarker-guided approaches. Simply replicating the successful model from breast cancer is not feasible.

#### 4.2.3. Urothelial Carcinoma

Urothelial Carcinoma (UC) has a very poor prognosis after developing resistance to platinum-based chemotherapy. ADC drugs, particularly those targeting Nectin-4 and TROP2, have become important pillars in later-line and even first-line treatment. Enfortumab Vedotin (EV) consists of a fully human anti-Nectin-4 antibody conjugated via a cleavable linker to the microtubule disruptor MMAE. Nectin-4 is highly expressed in many UCs (approximately 60%) but has limited expression in normal tissues, making it an ideal ADC target [162]. EV’s approval was first based on later-line therapy studies. EV-201 Cohort 1 and EV-301 studies established EV’s standard status in locally advanced or metastatic UC previously treated with platinum chemotherapy and a PD-1/PD-L1 inhibitor. In EV-201, the ORR was 44%, with a median DOR of 7.6 months [163,164]. The subsequent EV-301 Phase III study further confirmed that compared to chemotherapy, EV significantly improved median OS (12.9 months vs. 9.0 months) and PFS in these patients [165].

Even more landmark is the combination of EV with ICIs. The EV-302/KEYNOTE-A39 Phase III study compared EV combined with pembrolizumab versus standard chemotherapy in previously untreated advanced UC patients. Results showed that the combination group achieved significant improvements in both median PFS (12.5 months vs. 6.3 months) and OS (31.5 months vs. 16.1 months), with an ORR as high as 67.7% [166]. This study established a successful paradigm for combining ADCs with ICIs. Patient-reported outcomes analysis further confirmed that this combination regimen, while significantly improving survival, did not compromise patients’ quality of life and even brought clinically meaningful improvements in quality of life for patients with moderate-to-severe baseline pain [167]. Urothelial carcinoma has established a successful paradigm for ADC combination with ICIs as a new standard for first-line therapy. The single-agent activity of EV has demonstrated the effectiveness of the MMAE payload in this disease. The synergistic effect observed with its combination with pembrolizumab, far exceeding mere additive effects, likely stems from MMAE-induced immunogenic cell death (ICD) remodeling the TME and enhancing sensitivity to anti-PD-1 therapy. This success reveals the immunomodulatory potential of ADCs beyond direct cytotoxicity. It strongly suggests that future clinical development of ADCs, particularly for immunologically “cold” tumors or those with a highly immunosuppressive microenvironment, should prospectively explore combinations with immunotherapies.

Sacituzumab Govitecan (SG) has also shown some antitumor activity in UC. The Phase II IMMU-132-01 basket trial and the TROPICS-03 study both observed responses to SG in advanced UC patients [168]. However, in the dedicated Phase III TROPICS-04 study for UC, although SG showed a numerical improvement in ORR compared to chemotherapy (23% vs. 14%), the primary endpoint of OS did not reach statistical significance (median OS: 10.3 months vs. 9.0 months, HR = 0.86) [169]. This suggests that the value of SG in UC further exploration, possibly requiring biomarker selection to identify the benefiting population. In contrast, the failure of SG to demonstrate a clear improvement in overall survival (OS) in UC serves as a reminder that not every ADC design effective in one cancer type is readily transferable to another. The biological significance of TROP2 in UC, intratumoral drug release efficiency, or microenvironmental factors may constitute limitations.

#### 4.2.4. Gynecological Cancers

##### Cervical Cancer

Tisotumab Vedotin (TV) is the first ADC approved for recurrent or metastatic cervical cancer, targeting TF. TF expression is significantly higher in cervical cancer tissue compared to normal tissue, and its high expression is associated with advanced clinical stage and lymph node metastasis [170]. Its approval was based on the innovaTV 204/GOG-3023/ENGOT-cx6 Phase II single-arm study. This study enrolled 101 patients who had progressed after up to two prior systemic therapies (must include platinum-based chemotherapy). Results showed an ORR of 24% (including 7% complete responses), with a median DOR of 8.3 months [170]. The subsequent confirmatory Phase III innovaTV 301 study demonstrated that in the second- or third-line setting, TV compared to chemotherapy significantly prolonged OS (11.5 months vs. 9.5 months) and PFS (4.2 months vs. 2.9 months), reducing the risk of death by 30% [171]. A Phase II study explored TV combinations with bevacizumab, pembrolizumab, or carboplatin. Results showed that in both treatment-naive and pretreated recurrent or metastatic cervical cancer (r/mCC) patients, these combinations demonstrated encouraging antitumor activity and manageable toxicity, particularly the first-line TV plus carboplatin regimen achieving an ORR of 54.5%, providing new rationale and basis for frontline therapy [172]. Common adverse reactions to TV include ocular events, alopecia, and peripheral neuropathy, requiring corresponding prophylactic measures and close management [170,173]. The approval of TV demonstrates that in cervical cancer, which lacks driver mutations and has limited treatment options, targeting a non-canonical antigen like TF—which is associated with tumor progression—can also yield meaningful clinical benefit. This expands the scope of target selection for ADCs to include pan-cancer antigens linked to invasion and metastasis. Studies combining TV with chemotherapy, bevacizumab, or pembrolizumab have shown higher response rates, reaffirming the potential of ADCs as a cornerstone for combination therapies.

##### Ovarian Cancer

Ovarian cancer is highly heterogeneous, and ADCs targeting various antigens are being explored in this difficult-to-treat disease. Folate Receptor Alpha (FRα) is highly expressed in high-grade serous ovarian cancer and is an important therapeutic target. Mirvetuximab Soravtansine (MIRV) is an ADC targeting FRα, with the maytansinoid derivative DM4 as its payload. The key SORAYA Phase II study in platinum-resistant ovarian cancer showed that in patients with high FRα expression, MIRV achieved a confirmed ORR of 32.4% and a median DOR of 6.9 months, leading to its accelerated approval by the FDA [174]. Subsequent Phase III trials further confirmed the PFS and OS benefit of MIRV compared to chemotherapy, consolidating its position in platinum-resistant ovarian cancer. Moreover, MIRV has shown potential in platinum-sensitive ovarian cancer. A Phase II study evaluated MIRV as third-line or later therapy for FRα-positive platinum-sensitive ovarian cancer, achieving a high ORR of 51.9%, and even in the PARP inhibitor-resistant subgroup, the ORR remained at 45.8% [175].

Beyond FRα, ADCs targeting other antigens are under active development. For example, Mesothelin is expressed in most ovarian cancers, and its targeting ADC such as DMOT4039A is being evaluated in early clinical trials [176]. Additionally, ADCs targeting Sodium-dependent Phosphate Transporter 2b, B7-H4, and HER2 (expressed in some ovarian cancer subtypes) are also undergoing preclinical and clinical exploration in ovarian cancer, showing potential therapeutic prospects [177,178,179]. Histopathological detection, including IHC and companion diagnostics, is crucial for accurately screening patients who may benefit from these ADC treatments [180].

In ovarian cancer, particularly for platinum-resistant patients, ADCs represent a crucial therapeutic modality addressing unmet clinical needs. The success of MIRV in patients with high FRα expression validates the precision strategy of strictly relying on biomarker enrichment in highly heterogeneous tumors. However, the heterogeneity of FRα expression itself remains a major limitation for its efficacy. This raises a pivotal dilemma: for diseases like ovarian cancer that lack a universally high-expressing, ideal target, should the focus remain on identifying and enriching for increasingly niche biomarker-positive populations, or should efforts pivot towards developing ADCs with potent bystander effects capable of overcoming heterogeneity? Currently, both paths are being pursued in parallel. In the future, multi-omics profiling based on tissue or liquid biopsies may determine the optimal ADC therapeutic strategy for a broader patient population.

#### 4.2.5. Non-Small Cell Lung Cancer

Non-small cell lung cancer (NSCLC) has a complex spectrum of driver genes. ADCs provide new options for patients without traditional driver gene mutations or those resistant to targeted/immunotherapy. Approximately 2–4% of NSCLC harbor HER2 (ERBB2) kinase domain mutations, for which effective targeted therapies were previously lacking [181]. T-DXd has made a breakthrough in this area. The HER2-mutant cohort of a Phase II study showed that T-DXd achieved a confirmed ORR of 57.7%, median PFS of 10.1 months, and median OS of 17.8 months [146]. This efficacy led to the approval of T-DXd for HER2-mutant advanced NSCLC in many countries worldwide, filling a therapeutic gap. The ADC SHR-A1811 (Trastuzumab Rezetecan) also showed high activity in HER2-mutant NSCLC. A Phase II study reported a confirmed ORR as high as 73% in pretreated Chinese patients [182]. Its global Phase I trial also showed broad prospects and manageable toxicity in various HER2-expressing or mutant solid tumors [183].

TROP2 is expressed in most NSCLCs and is another promising target. Dato-DXd was compared head-to-head with docetaxel in the Phase III TROPION-Lung01 study for pretreated advanced or metastatic NSCLC patients. Results showed that Dato-DXd significantly improved PFS (median PFS: 4.4 months vs. 3.7 months, HR = 0.75), with more pronounced benefit in the non-squamous subgroup (HR = 0.63). Although OS did not show a statistical difference, its safety profile was more favorable, with a lower incidence of ≥grade 3 treatment-related adverse events (25.6% vs. 42.1%), but attention to ILD (incidence 8.8%) and stomatitis is needed [184]. The higher incidence of stomatitis observed clinically with Dato-DXd suggests its payload, DXd, may have a significant impact on rapidly proliferating oral mucosal epithelial cells. This constitutes a differentiated toxicity profile from the interstitial lung disease (ILD) associated with T-DXd. This divergence in organ-specific toxicity underscores the important role played by the tissue distribution specificity determined by the antibody moiety in shaping the final toxicity profile of an ADC. Consequently, clinical development necessitates tailored prophylactic and supportive care strategies for the unique toxicities of different ADCs. Furthermore, in patients with oncogenic driver gene alterations like EGFR who failed targeted therapy and chemotherapy, the Phase II TROPION-Lung05 study showed an ORR of 35.8% for Dato-DXd, indicating its potential in later-line therapy [185].

In a Phase III study, Sacituzumab Govitecan (SG) did not significantly improve OS compared to docetaxel in the overall population of pretreated metastatic NSCLC. However, a trend towards OS benefit (HR = 0.75) was observed in the subgroup with no response (stable disease or progression) to prior immunotherapy, offering a potential treatment option for this population [186]. For non-squamous NSCLC with c-MET protein overexpression, Telisotuzumab Vedotin has shown potential. The Phase II LUMINOSITY trial in pretreated, c-Met overexpressing, EGFR wild-type patients showed an overall ORR of 28.6%, with an ORR of 34.6% in c-Met high expressors, and a median PFS of 5.7 months [187]. This provides a new targeted treatment option for this specific population.

In NSCLC, T-DXd perfectly exemplifies the potential of ADCs as tumor-agnostic targeted therapies. Its mechanism relies not on HER2 overexpression but on the normal internalization of the mutant protein, which differs from the HER2-overexpression paradigm in breast cancer. On the other hand, comparison studies of Dato-DXd and SG versus docetaxel in later-line treatment of advanced NSCLC suggest that for widely expressed targets at moderate levels, such as TROP2, ADC monotherapy may have reached an efficacy plateau in this heavily pretreated, highly heterogeneous population. Future breakthroughs will likely depend on more precise biomarkers and frontline combinations with immunotherapy or targeted therapy, aiming to exert maximal effect when the disease is at an earlier stage with lower heterogeneity. Table 2 summarizes the current phase III clinical studies of relevant ADC drugs in cancer.

## 5. Core Challenges Facing Current ADCs

### 5.1. Complexity of Target Expression and Efficacy Prediction

The traditional view holds that high expression of the target antigen is a key predictor of ADC efficacy. For example, Mirvetuximab Soravtansine and Telisotuzumab Vedotin both showed better efficacy in patients with high target expression [174,187]. However, this linear relationship is being challenged. The efficacy of Tisotumab Vedotin (anti-TF) shows no clear correlation with TF expression levels [172]. Similarly, clinical responses to BV and Polatuzumab Vedotin are also independent of target expression levels [8,188]. The reasons for this deviation are multifaceted. First, ADCs using cleavable linkers and hydrophobic payloads (like T-DXd’s DXd and SG’s SN-38) can, after killing the target cell, release payload that acts on neighboring tumor cells, thereby reducing absolute dependence on target expression [147,189]. Furthermore, detection technologies have limitations. Except for HER2, most targets lack standardized detection protocols. Taking Trop-2 as an example, the ASCENT and TROPICS-02 trials used different H-score criteria, leading to differing conclusions [148,189]. Immunohistochemistry (IHC) only reflects local antigen status in the biopsied area and cannot capture heterogeneity across metastatic sites or spatiotemporal dynamics of antigen distribution [190]. Additionally, target biology is tumor-type specific; the same target can play different roles in different cancers. HER2-low benefits from T-DXd in breast cancer but has minimal efficacy in gastric cancer, possibly due to differing tissue sensitivity to the payload [8,191]. For targets like TROP2 with spatiotemporal heterogeneity in expression, the current reliance on single-site biopsy-based immunohistochemistry (IHC) as a companion diagnostic has fundamental limitations. IHC cannot capture systemic heterogeneity and struggles to reflect the functional activity of the antigen; while functional imaging techniques like immuno-PET can provide whole-body, quantitative distribution information, they face challenges related to cost and accessibility [190,192]. Therefore, the most viable future path involves developing a layered, integrated strategy. This entails first promoting the standardization and quantification of IHC assays, establishing them as the cornerstone for broad screening. For complex or challenging cases, immuno-PET should then be employed as a supplementary tool to assess whole-body target distribution and functional status. Ultimately, precision therapy with ADCs must evolve towards a multidimensional assessment framework that integrates morphological, molecular, and functional imaging.

### 5.2. Intratumoral Distribution and Low Delivery Efficiency

The ideal delivery of ADCs faces multiple physiological barriers. Studies show that only about 0.1–2% of the injected dose ultimately accumulates at the tumor site [192]. ADCs in circulation can bind to targets or Fc receptors expressed on normal tissues, getting “sequestered” in off-target organs, reducing the drug amount available to the tumor [193]. High-affinity binding of the antibody to the target leads to rapid internalization on the surface of perivascular tumor cells, hindering deep penetration of the ADC into the tumor and causing uneven distribution [194]. Moreover, spatiotemporal heterogeneity and downregulation of target expression allow some tumor cells to escape ADC killing, becoming a source of resistance [2]. Antibody affinity requires a balance between efficient internalization and deep tumor penetration. Preclinical studies suggest that a moderately high affinity in the nanomolar range (~0.1–5 nM) may be optimal, enabling both effective target binding/internalization and permitting some antibody dissociation for deeper tumor diffusion [195]. However, most current FDA-approved ADCs (e.g., T-DM1 and T-DXd based on trastuzumab) directly inherit the high affinity (Kd ~0.1 nM) of their parental naked antibodies, originally designed for target blockade rather than optimized drug delivery. Consequently, no approved ADC systematically demonstrates affinity optimized for penetration. This highlights a crucial future design direction: treating affinity as a tunable variable or employing strategies like bispecific antibodies to cooperatively achieve both high-efficiency internalization and deep penetration.

### 5.3. The Balancing Act in Linker Design

The linker must remain absolutely stable in the bloodstream while efficiently releasing the payload inside tumor cells; this contradiction is the core challenge in design. Theoretically, highly stable linkers (like the non-cleavable linker used in T-DM1) can reduce off-target toxicity but may limit efficacy due to insufficient payload release, especially in tumors with low antigen expression or inefficient internalization [196]. Conversely, linkers with some “leakage” characteristics (like the tetrapeptide linker in T-DXd), while leading to higher systemic free payload concentrations, confer a powerful bystander effect that enable performance in HER2-low tumors [8]. Furthermore, overly stable linkers (especially those producing homogeneous ADCs via site-specific conjugation) may deliver more intact ADC to normal tissues expressing the target, causing “on-target, off-tumor” toxicity. For example, studies found that ARX788 (using a non-cleavable linker) and DCDS0780A (a site-specific anti-CD79b ADC) were both halted in clinical development due to severe ocular toxicity and other on-target toxicities, whereas the slightly less stable Polatuzumab Vedotin was successfully approved [197,198]. The phenomenon of severe “on-target, off-tumor” toxicity with highly stable ADCs arises from the interplay between the target expression profile and the ADC’s pharmacokinetic properties, rather than a single determining factor. Cases like ARX788 and DCDS0780A demonstrate that for targets with low-level expression in critical normal tissues (e.g., cornea), employing non-cleavable, highly stable linkers and a homogeneous, high DAR design can lead to efficient internalization and accumulation of the intact ADC in normal tissues, thereby exacerbating organ-specific toxicity [197,198]. This suggests that a certain degree of controlled linker release or a faster systemic clearance rate might act as a pharmacological safety valve by reducing long-term accumulation in normal tissues. Therefore, the ideal stability of an ADC linker is not absolute but should be contextually designed based on the target’s biology. For targets absent in critical organs, maximal stability should be pursued. For targets with low-level expression, a balance between stability and normal tissue exposure risk must be struck, or conditional activation mechanisms such as prodrugs should be introduced.

### 5.4. Toxicity Profile and Resistance

The toxicity profile of ADCs is complex. First, there is the platform toxicity of the payload, related to the mechanism of action of the small molecule cytotoxin itself. For instance, tubulin inhibitors (MMAE, DM1) often cause neutropenia and peripheral neuropathy; whereas DNA damaging agents are associated with increased risk of thrombocytopenia, anemia, and ILD [199,200]. MMAF is particularly notable for ocular toxicity due to its specific metabolic pathway. Second, there is “on-target, off-tumor” toxicity, caused by low-level expression of the target antigen in normal tissues. For example, Trop-2 is expressed in the skin, cornea, and gastrointestinal tract, leading to rash, dry eye, and diarrhea with Trop-2 targeting ADCs like Sacituzumab Govitecan [201]; while HER2-targeting ADCs may affect HER2-expressing cardiomyocytes, although clinically significant cardiotoxicity is relatively rare. ADCs also have unique adverse reactions whose mechanisms may be related to the overall ADC design. The most typical is T-DXd-related ILD. Although its exact mechanism is not fully elucidated, it has become the most critical adverse event requiring vigilance and management in the clinical use of this drug [202]. Additionally, the tendency of ADCs to aggregate may lead to non-specific uptake by immune cells via Fcγ receptors, causing unintended cytokine release and hepatotoxicity, among others [203].

Resistance mechanisms are complex and diverse, greatly limiting the long-term efficacy of ADCs. The main mechanisms include: ① Target-related mechanisms: These include downregulation, mutation, or complete loss of the target antigen, which is the most direct way for tumor cells to evade ADC attack [204]. ② Impairment of ADC internalization and intracellular transport: Even if the ADC successfully binds the cell surface antigen, inefficient internalization or post-internalization routing into non-degradative pathways can prevent effective payload release [49]. ③ Lysosomal function defects: Abnormal lysosomal pH, reduced protease (cathepsin B) activity, or dysfunctional transport proteins can all hinder effective linker cleavage and payload release [205]. ④ Payload resistance mechanisms: Tumor cells can upregulate drug efflux pumps to expel released cytotoxins; or directly develop resistance to the payload by mutating the drug target or enhancing DNA repair capacity [204,206]. Notably, tumor heterogeneity is a core factor driving resistance. Spatiotemporal heterogeneity in target expression prevents ADCs from killing all tumor cells, ultimately leading to clonal selection and treatment failure [2]. Furthermore, sequential use of ADCs with the same or similar payload mechanisms can easily lead to cross-resistance, whereas switching payload mechanisms has proven to be an effective clinical strategy to overcome resistance [207,208]. In the future, combination with drugs targeting specific resistance mechanisms is a promising direction for exploration.

Regarding the priority of switching targets versus payload classes in ADC sequential therapy, there is currently a lack of direct head-to-head Phase III trial evidence. However, based on existing research, it may be concluded that when target expression (e.g., HER2) is preserved, prioritizing a switch to a payload with a different mechanism of action is a key strategy to overcome payload-driven resistance. For instance, the DESTINY-Breast02 study confirmed that patients resistant to T-DM1 (a microtubule inhibitor) could still achieve significant efficacy by switching to T-DXd (a topoisomerase I inhibitor) [8,143]. If target downregulation or loss occurs, switching the target antigen should be prioritized. Clinical decision-making must be based on an individualized assessment of the resistance mechanism, integrating drug accessibility and toxicity profiles. Future precision sequential strategies await establishment through biomarker-driven prospective studies.

### 5.5. Economic and Accessibility Challenges

The high price of ADCs impacts patient access. Their high cost stems from multiple factors. First, research and development is expensive, with resources and time required for everything from target discovery, antibody engineering, and linker-payload optimization to complex preclinical and clinical studies [209]. Second, the manufacturing process is extremely complex. ADC production involves large-scale cell culture for antibodies, safe handling of highly potent toxins, complex conjugation reactions (especially with high demands for site specificity and homogeneity), and rigorous quality control, all of which drive up production costs [210,211]. Finally, market pricing strategies also reflect their value as breakthrough therapies and monopolistic pricing during the patent protection period.

This high cost directly translates into high treatment expenses, placing a heavy burden on healthcare payment systems and patient out-of-pocket costs. Accessibility to ADCs varies greatly across different countries and regions due to differences in economic development levels [212]. Even in developed countries, payers face difficult choices. To improve accessibility, promoting the development of biosimilars to introduce market competition, optimizing production processes to reduce costs, or exploring more cost-effective dosing regimens are potential strategies [209,213]. Addressing the economic challenges of ADCs is a key step towards realizing their value as tumor “missiles” for the benefit of all.

## 6. Innovative Directions for Next-Generation ADC Technology

Next-generation ADC technologies are rapidly evolving towards the goals of enhancing efficacy, overcoming resistance, and expanding the therapeutic window. These strategies exhibit significant differences in innovativeness, technical complexity, and clinical translation maturity. Based on existing preclinical evidence and early clinical trial data, we can perform a preliminary priority assessment of these emerging platforms: among them, bispecific ADCs and combination strategies enabled by site-specific conjugation technologies have entered a phase of broad clinical validation; novel linkers (e.g., microenvironment-responsive) and novel payloads (e.g., protein degraders) are in a transitional phase from proof-of-concept to early clinical testing; while platforms based on nanotechnology or complex external control (e.g., photo-control) remain largely in the preclinical exploration stage. The following discussion will be stratified according to their likelihood of nearing clinical translation.

### 6.1. Innovation in Antibodies and Targeting Strategies

#### 6.1.1. Bispecific ADCs

These ADCs can simultaneously bind two different tumor-associated antigens or one tumor antigen plus an immune cell marker [214]. BsADCs can enhance internalization by inducing receptor clustering or overcome tumor heterogeneity through dual-target engagement. BL-B01D1 has shown high objective response rates in heavily pretreated EGFR-mutant NSCLC patients, demonstrating great potential [214]. Advances in bioorthogonal chemistry, particularly the application of click chemistry, provide efficient, mild, and specific tools for constructing such complex bifunctional conjugate systems, ensuring product homogeneity and stability [215]. Among the many innovative targeting strategies, bispecific ADCs (BsADCs) are currently the closest to widespread clinical translation and are already supported by substantial clinical evidence. Their design logic directly addresses the core challenges of first-generation ADCs—target heterogeneity and insufficient internalization efficiency—and can be built upon relatively mature antibody engineering and conjugation platforms. For example, the Phase I study of BL-B01D1, which targets EGFRxHER3, demonstrated high objective response rates in various solid tumors, confirming the feasibility of this strategy [214]. It is anticipated that more BsADCs will enter pivotal clinical studies in the foreseeable future.

#### 6.1.2. Prodrug ADCs (Pro-ADCs)

In Pro-ADCs, the antibody portion is masked by a peptide, rendering it inactive in healthy tissues. This peptide is cleaved only by specific proteases present in the tumor microenvironment, exposing the antibody binding domain and thereby activating the ADC, aiming to minimize on-target, off-tumor toxicity [216]. Although early candidates did not meet expectations regarding efficacy and toxicity control, this remains an important exploratory direction [217]. In contrast, the clinical translation of Propodi-drug conjugates (Pro-ADCs) faces greater challenges and currently remains a highly innovative yet insufficiently clinically validated strategy. Although the concept of “tumor microenvironment activation” is highly attractive for minimizing off-target toxicity, clinical results from early candidates like CX-2009 indicate that achieving efficient, specific, and consistent protease activation within the complex human tumor microenvironment remains a significant bottleneck. The success of this strategy may depend on a more precise understanding of specific tumor proteaseomes and breakthroughs in corresponding linker chemistry. Its prospects for broad clinical application await validation through more candidate drugs.

#### 6.1.3. Novel Antibody Formats and Co-Administration

Using small formats like nanobodies or single-domain antibodies can improve tumor penetration [218]. Furthermore, preclinical studies show that co-injecting unconjugated “naked antibodies” or fast-clearing competitive inhibitors can block peripheral targets and promote deeper penetration of ADCs into tumors [219,220]. These strategies are currently primarily in the preclinical or early proof-of-concept stage. Their generalizability as platform technologies still requires validation across more targets and tumor models. Furthermore, the pharmacokinetic challenges associated with small-format antibodies necessitate additional solutions.

### 6.2. Upgrades in Linker and Conjugation Technology

Next-generation linker design moves beyond the binary choice of “stable or not,” instead pursuing precise control of payload release in specific spatiotemporal contexts. Simultaneously, novel conjugation strategies provide a solid foundation for achieving this goal.

#### 6.2.1. TME-Responsive Linkers

Utilizing physiological signals unique to the TME, such as hypoxia, high levels of reactive oxygen species (ROS), or specific enzymes, to design “AND-gate” logic control. For example, hypoxia-sensitive azobenzene linkers remain stable under normal tissue oxygen levels but are reduced and cleaved upon entering the hypoxic tumor environment, releasing highly toxic drugs and effectively reducing non-targeted toxicity [221]. ROS-responsive diazaborine linkers are also in preclinical development [222].

#### 6.2.2. Photo-Controlled Release and Amart-Responsive ADCs

Using light as an external controllable stimulus, ADC is endowed with excellent spatio-temporal regulation ability. Near-infrared (NIR) light-controlled systems are more ideal excitation sources due to their deeper tissue penetration and lower biotoxicity [223,224]. For instance, dual conjugation of IR700 with a non-cleavable ADC (like T-DM1) constructs a composite ADC that, upon NIR light irradiation, achieves efficient drug release and produces a photo-mediated bystander effect, overcoming tumor antigen heterogeneity [63]. Such photo-controlled ADCs offer precise treatment options for superficial tumors [225].

#### 6.2.3. Bioorthogonal Controlled Release and Novel Conjugation Strategies

Bioorthogonal chemistry allows for “on-demand” activation and release of drugs in vivo. For example, the “click-to-release” strategy uses the inverse electron demand Diels–Alder reaction between trans-cyclooctene and tetrazine for precise activation via a two-step administration [226]. Furthermore, enzymatic conjugation and non-natural amino acid incorporation enable site-specific conjugation, producing homogeneous, stable ADCs, and providing a platform for precise loading of complex payloads [38,227]. These methods overcome the product heterogeneity issues associated with traditional lysine or cysteine conjugation.

#### 6.2.4. Pharmacokinetic Module Optimization

Introducing hydrophilic modules like PEG can counteract the aggregation tendency caused by hydrophobic payloads, improving ADC solubility and in vivo half-life. However, the degree of PEGylation needs careful balancing to avoid excessively affecting payload membrane permeability and the bystander effect [228].

Within the field of linkers and conjugation technologies, the maturity of different strategies varies significantly. Site-specific conjugation technologies (e.g., AJICAP, THIOMAB) have become the new industry standard, widely adopted in constructing next-generation ADCs, with their advantages in improving homogeneity, pharmacokinetics, and efficacy clinically confirmed. Microenvironment-responsive linkers (e.g., hypoxia-, ROS-responsive) are a cutting-edge focus in linker design, but most remain in the preclinical development stage, and their stability and response efficiency in humans await validation. Technologies such as photo-controlled release and bioorthogonal “click-to-release,” while demonstrating unparalleled potential for spatiotemporal control, face clinical translation hurdles due to device complexity, tissue penetration depth, and potential immunogenicity, currently placing them in a more exploratory, long-term direction.

### 6.3. Diversification and Platform Expansion of Payload Mechanisms

#### 6.3.1. Immune-Stimulating Antibody Conjugates (ISACs)

ISACs replace the traditional cytotoxic payload with Toll-like receptor (TLR) or STING agonists. They locally activate the innate immune system within the tumor, remodel the immune microenvironment, theoretically converting “cold” tumors to “hot,” and inducing immune memory [229]. However, the clinical translation of ISACs faces challenges, mainly due to the narrow therapeutic window of agonists, easily triggering systemic inflammatory responses. BDC-1001 (TLR7/8 agonist) showed limited efficacy, while XMT-2056 (STING agonist) had its clinical trial suspended due to serious adverse events, highlighting the difficulty in managing their safety [230]. ISACs represent a bold attempt to shift the paradigm of ADCs from direct cytotoxicity to immune activation, yet their clinical translation has met significant setbacks, highlighting the high-risk nature of this strategy. Employing potent immune-stimulating molecules like TLR or STING agonists as payloads presents a narrow therapeutic window. The limited monotherapy activity of BDC-1001 and the clinical hold for XMT-2056 due to serious adverse events both indicate that controlling systemic immune activation is a major challenge. The success of ISACs may depend on stricter mechanisms for tumor-localized activation or the use of more tempered agonists. Currently, this field is at a critical juncture of re-evaluation and design optimization.

#### 6.3.2. Immunomodulatory ADCs

Beyond directly killing tumor cells, growing evidence suggests that traditional cytotoxic ADCs also have important immunomodulatory functions. They can induce ICD, releasing damage-associated molecular patterns like HMGB1 and ATP, thereby activating antitumor immune responses [231]. For example, an ADC targeting GPC2 can remodel the tumor microenvironment, promoting the migration of macrophages and T cells to the tumor site. Combining such ADCs with ICIs can potentially synergize by increasing tumor antigen release and presentation, enhancing tumor sensitivity to ICIs [231,232]. This adds a new dimension to ADC efficacy.

#### 6.3.3. Degrader–Antibody Conjugates (DACs)

DACs replace the payload with proteolysis-targeting chimeras (PROTACs), leveraging the intracellular ubiquitin-proteasome system to selectively degrade target proteins. This is particularly promising for traditionally “undruggable” transcription factors or mutant oncoproteins. ORM-5029 (targeting HER2, degrading GSPT1) showed efficacy comparable to T-DXd in preclinical studies and has entered clinical stages [233]. The immunomodulatory role of traditional ADCs via the ICD is widely recognized and is being actively validated in combination with immunotherapies. DACs, on the other hand, combine the targeted delivery advantage of ADCs with the protein degradation mechanism of PROTACs, holding promise for targeting ‘undruggable’ proteins. Pioneering DACs like ORM-5029 have entered Phase I clinical trials. Preliminary clinical data will be crucial for assessing their efficacy and unique toxicity profiles. If early clinical validation is successful, DACs could become an important new drug class in the coming years.

#### 6.3.4. Radionuclide Drug Conjugates (RDCs)

RDCs conjugate antibodies with radionuclides (^177^Lu), integrating diagnosis and therapy. Their killing mechanism does not rely on internalization and has a crossfire effect, able to kill neighboring heterogeneous tumor cells. The successful approval of Pluvicto (anti-PSMA) and Lutathera (anti-SSTR) validates the feasibility of this platform, but its development is limited by the scarcity of highly selective targets [234]. Thus, from a certain perspective, RDCs are no longer a “next-generation” technology but a mature, clinically translated platform. The approvals of Pluvicto and Lutathera [234] validate the immense value of this platform in cancers with limited therapeutic options. The primary bottleneck for its further development lies not in the technology itself, but in the discovery of ideal targets—like PSMA and SSTR—that exhibit exceptionally high tumor selectivity with minimal expression in normal tissues. Future growth will depend on the deep integration of nuclear medicine and tumor biology in target discovery.

#### 6.3.5. Multi-Payload ADCs

To overcome tumor heterogeneity and multiple resistance mechanisms, the design of dual-payload and multi-payload ADCs is a research hotspot. By covalently linking two (or more) drugs with different mechanisms of action (microtubule inhibitor MMAF and DNA damaging agent SN-38) onto the same antibody, synergistic or additive antitumor activity can be achieved, effectively killing cells resistant to a single payload, such as non-dividing cells [235]. Site-specific conjugation technologies like AJICAP allow flexible control over the ratio and type of different drugs loaded onto a single antibody, forming homogeneous dual-payload ADCs, even exceeding traditional DAR ranges (up to DAR 10), further broadening the design space [236,237]. Multi-payload ADC architectures like SPARC enable synchronized delivery of different drugs, achieving synergistic release in vivo and enhancing tumor cell killing [44]. In models of breast cancer, triple-negative breast cancer, etc., dual-payload ADCs have shown superior efficacy compared to single-payload ADCs by targeting multiple signaling pathways [238,239]. The key technical challenges for multi-payload ADCs lie in ensuring chemical compatibility between different payloads, their stable conjugation, and coordinated in vivo release. Homogeneous dual-payload ADCs constructed using technologies like AJICAP have advanced into late-stage preclinical research. Although no product has been approved yet, this strategy is highly anticipated for its potential to address core clinical challenges, and it is expected that more candidates will enter clinical trials in the foreseeable future.

### 6.4. Integration of Nanotechnology and ADCs

The introduction of nanotechnology provides innovative solutions for ADC development. Novel nano-platforms such as antibody-DNA nanostructures and polymer/lipid nanocarriers can enhance drug loading capacity and achieve controlled release and precise drug delivery through nanostructure design. For example, DNA nanotubes modified with lipids or cholesterol can enhance binding to cancer cell membranes, improving cellular uptake [240,241]. Nanocarriers can also be multifunctionally modified to simultaneously deliver cytotoxic drugs and immunomodulatory molecules, activating tumor-associated immune responses and combining tumor cell killing with immune activation [242]. Photoimmunotherapy (PIT), which combines photodynamic therapy with ADCs, is a typical example of the fusion of nanotechnology and ADCs [243]. The integration of nanocarriers with ADCs constitutes a highly interdisciplinary frontier that remains predominantly in the preclinical exploration stage. These platforms hold the promise of achieving exceptionally high drug loading capacity, co-delivery of multiple drugs, and sophisticated release logics far beyond those of traditional ADCs. However, their translation to the clinic faces a series of additional challenges, including the complexities of scalable manufacturing, the intrinsic biodistribution and potential toxicity of the nanomaterials themselves, and uncertainties in the regulatory pathway. While specific modalities like PIT have been approved for superficial tumors, establishing nanotechnology as a universal platform for ADC enhancement will require fundamental breakthroughs in both safety and manufacturing feasibility. Table 3 presents a summary of current studies on next-generation ADC drugs. To provide a clearer overview of the ADC landscape evolution, Table 4 summarizes and contrasts the key features of clinically validated platforms with those of promising emerging technologies.

## 7. Conclusions

Currently, ADCs are an important class of cancer treatments, with several FDA-approved ADCs available for treating various cancers. Despite all the advances in cancer treatment, inherent and acquired resistance remains a major obstacle to successful treatment. ADCs are evolving from simple “smart chemotherapy” into modular platforms. Bispecific antibodies, smaller antibody forms, and combination dosing strategies can enhance drug penetration. Future work will focus on sequential therapies and combination regimens that address resistance, as well as manufacturing/dosing strategies that improve drug affordability. These initiatives will work together to transform ADCs into more reliable treatment options, benefiting numerous patients.

## Figures and Tables

**Table 1:** FDA-approved ADCs. Date from (www.fda.gov), search time: 30 October 2025.

Generic Name	Brand	Target Antigen	Payload	Linker (Type)	Initial FDA Approval (Date)	Key Label Milestones (Date → Note)	Primary U.S. Indications (Summary)
Gemtuzumab ozogamicin	Mylotarg	CD33	Calicheamicin	Acid-labile hydrazone (cleavable)	17 May 2000	1 September 2017 → U.S. re-approval with modified dose/schedule	CD33-positive AML (adult ± pediatric; label-specific)
Brentuximab vedotin	Adcetris	CD30	MMAE (auristatin E)	Val-Cit–PABC (protease-cleavable)	19 August 2011	11 September 2017 → regular approval/expanded indications	CD30-expressing lymphomas (cHL, sALCL; other CD30+ PTCLs)
Ado-trastuzumab emtansine	Kadcyla	HER2	DM1 (maytansinoid)	MCC thioether (non-cleavable)	22 February 2013	6 May 2019 → adjuvant EBC approval	HER2-positive breast cancer (various settings)
Inotuzumab ozogamicin	Besponsa	CD22	Calicheamicin	Acid-cleavable	17 August 2017	—	R/R B-cell precursor ALL (adults)
Moxetumomab pasudotox-tdfk	Lumoxiti	CD22	Pseudomonas exotoxin (recombinant immunotoxin)	Protein fusion (non-cleavable)	13 September 2018	July 2023 → U.S. market withdrawal (sponsor decision)	R/R hairy cell leukemia after ≥2 prior systemic therapies (incl. PNA)
Polatuzumab vedotin-piiq	Polivy	CD79b	MMAE	Val-Cit–PABC (protease-cleavable)	10 June 2019	19 April 2023 → approval in previously untreated DLBCL (R-CHP)	DLBCL (in combination regimens)
Enfortumab vedotin-ejfv	Padcev	Nectin-4	MMAE	Val-Cit–PABC (protease-cleavable)	18 December 2019	9 July 2021 → conversion to full (regular) approval + expanded indications	Locally advanced/metastatic urothelial carcinoma (multiple settings)
Fam-trastuzumab deruxtecan-nxki	Enhertu	HER2	DXd (exatecan derivative; topo-I inhibitor)	Tetrapeptide-based cleavable	20 December 2019	11 August 2022 → HER2-mutant NSCLC (accelerated); 5 April 2024 → tumor-agnostic HER2-positive solid tumors (accelerated); 27 January 2025 → HER2-ultralow HR + MBC	Multiple HER2-expressing settings per label (breast incl. HER2-low/ultralow, NSCLC HER2-mutant, gastric/GEJ, tumor-agnostic)
Sacituzumab govitecan-hziy	Trodelvy	TROP2	SN-38 (topoisomerase I inhibitor)	CL2A (hydrolyzable)	22 April 2020	7 April 2021 → regular approval in mTNBC; 3 February 2023 → HR+/HER2- MBC expansion; 22 November 2024 → U.S. mUC indication withdrawn	mTNBC; HR+/HER2- metastatic breast cancer
Loncastuximab tesirine-lpyl	Zynlonta	CD19	PBD dimer (SG3199)	Protease-cleavable (val-ala; tesirine/SG3249)	23 April 2021	—	R/R large B-cell lymphoma after ≥2 prior lines
Tisotumab vedotin-tftv	Tivdak	Tissue factor (TF)	MMAE	Val-Cit–PABC (protease-cleavable)	20 September 2021	29 April 2024 → conversion to full approval	Recurrent/metastatic cervical cancer after chemotherapy
Mirvetuximab soravtansine-gynx	Elahere	FRα	DM4 (maytansinoid)	sulfo-SPDB (disulfide; cleavable)	14 November 2022	22 March 2024 → conversion to full approval	FRα-positive, platinum-resistant ovarian/fallopian tube/primary peritoneal cancer
Datopotamab deruxtecan-dlnk	Datroway	TROP2	DXd (exatecan derivative; topo-I inhibitor)	Tetrapeptide-based cleavable	17 January 2025	23 June 2025 → accelerated approval for EGFR-mutated NSCLC	HR+/HER2- metastatic breast cancer; EGFR-mutated NSCLC (accelerated)
Belantamab mafodotin-blmf	Blenrep	BCMA	MMAF (auristatin F)	Maleimidocaproyl (mc; non-cleavable)	5 August 2020 (original, withdrawn November 2022)	23 October 2025 → U.S. re-approval in combination with bortezomib + dexamethasone	R/R multiple myeloma (BVd combination; see label details)

HER2, human epidermal growth factor receptor 2 (ERBB2); FRα, folate receptor-alpha; BCMA, B-cell maturation antigen (TNFRSF17); TF, tissue factor (F3); TROP2, trophoblast cell-surface antigen 2 (TACSTD2); Nectin-4, poliovirus receptor-related 4 (PVRL4); EGFR, epidermal growth factor receptor; MMAE, monomethyl auristatin E; MMAF, monomethyl auristatin F; DM1, derivative of maytansine; DM4, derivative of maytansine; DXd, deruxtecan, an exatecan derivative; SN-38, active metabolite of irinotecan; PBD, pyrrolobenzodiazepine; calicheamicin, DNA-damaging enediyne; PE, Pseudomonas exotoxin; Val-Cit, valine–citrulline; PABC, para-aminobenzyloxycarbonyl; MCC, 4-(N-maleimidomethyl)cyclohexane-1-carboxylate; CL2A, 2-(2-chloroacetyl)-L-lysyl-L-leucyl linker (hydrolyzable); sulfo-SPDB, N-succinimidyl 4-(2-pyridyldithio)butanoate, sulfonated (cleavable disulfide); mc, maleimidocaproyl (non-cleavable); val-ala, valine–alanine (protease-cleavable dipeptide); AML, acute myeloid leukemia; ALL, acute lymphoblastic leukemia; DLBCL, diffuse large B-cell lymphoma; cHL, classical Hodgkin lymphoma; sALCL, systemic anaplastic large cell lymphoma; PTCL, peripheral T-cell lymphoma; NSCLC, non-small cell lung cancer; mTNBC, metastatic triple-negative breast cancer; HR+, hormone receptor-positive (ER and/or PR); HER2-, HER2-negative; MBC, metastatic breast cancer; mUC, metastatic urothelial carcinoma; GEJ, gastroesophageal junction. Treatment/Label shorthand—R/R, relapsed or refractory (prior-therapy setting); EBC, early breast cancer; PNA, purine nucleoside analog; BVd, bortezomib + dexamethasone; R-CHP, rituximab + cyclophosphamide + doxorubicin + prednisone.

**Table 2:** Details of selected ADC candidate drugs currently in phase III clinical trials (clinicaltrials.gov); search time: 30 October 2025.

Cancer		ADC Drug Name (Code)	Target	Primary Objective of Phase III Clinical Trial	Clinical Trial Number
Breast Cancer	HER2-Positive Breast Cancer	DP303c	HER2	To evaluate the efficacy and safety of DP303c compared to T-DM1 in patients with HER2-positive advanced breast cancer.	NCT06313086
	HER2-Positive Advanced Breast Cancer	DP303c	HER2	To evaluate the efficacy and safety of DP303c in patients with HER2-positive advanced breast cancer (comparator: trastuzumab, vinorelbine, or capecitabine).	NCT05901935
	HER2-Low, HR-Positive Metastatic Breast Cancer	DB-1303/BNT323	HER2	To evaluate the efficacy of DB-1303/BNT323 compared to investigator’s choice of chemotherapy in terms of PFS.	NCT06018337
	HER2-Positive Breast Cancer	ARX788	HER2	To compare the efficacy and safety of ARX788 combined with pyrotinib versus TCBHP (trastuzumab + pertuzumab + docetaxel + carboplatin) as neoadjuvant therapy.	NCT05426486
	HER2-Positive Advanced/Metastatic Breast Cancer	MRG002	HER2	To evaluate the efficacy and safety of MRG002 versus T-DM1 in patients with HER2-positive, unresectable locally advanced or metastatic breast cancer.	NCT04924699
	HR-Positive/HER2-Negative Breast Cancer	Dato-DXd	TROP2	To compare the safety and efficacy of Dato-DXd with investigator’s choice of standard chemotherapy in patients with inoperable or metastatic HR-positive/HER2-negative breast cancer.	NCT05104866
	HER2-Low, HR-Positive Breast Cancer	T-DXd	HER2	To compare the efficacy, safety, and tolerability of T-DXd with investigator’s choice of chemotherapy in patients with HER2-low, HR-positive breast cancer.	NCT04494425
Lung Cancer	Small Cell Lung Cancer	Ifinatamab deruxtecan (I-DXd)	B7-H3	To compare the efficacy and safety of I-DXd with physician’s choice of treatment in patients with relapsed small cell lung cancer.	NCT06203210
	EGFR-Mutant Non-Small Cell Lung Cancer	Patritumab Deruxtecan (HER3-DXd)	HER3	To compare the efficacy and safety of Patritumab Deruxtecan with platinum-based chemotherapy in patients with EGFR-mutant non-small cell lung cancer.	NCT05338970
	EGFR-Mutant Non-Small Cell Lung Cancer	SYS6010	EGFR	To evaluate the efficacy and safety of SYS6010 compared to platinum-based chemotherapy in patients with EGFR-mutant locally advanced or metastatic non-small cell lung cancer.	NCT06927986
	CEACAM5-Positive Non-Small Cell Lung Cancer	Tusamitamab ravtansine (SAR408701)	CEACAM5	To evaluate the progression-free survival and overall survival of Tusamitamab ravtansine compared to docetaxel in patients with CEACAM5-high metastatic non-squamous non-small cell lung cancer.	NCT04154956
Gastrointestinal Cancer	Metastatic Colorectal Cancer	Telisotuzumab Adizutecan (ABBV-400)	c-Met	To compare the adverse events and disease activity of Telisotuzumab Adizutecan with LONSURF (trifluridine/tipiracil) plus bevacizumab in patients with c-Met protein overexpressing refractory metastatic colorectal cancer.	NCT06614192
	Gastric or Gastroesophageal Junction Adenocarcinoma	AZD0901	CLDN18.2	To evaluate the efficacy and safety of AZD0901 compared to investigator’s choice of therapy as second-line or later treatment in patients with CLDN18.2-positive advanced or metastatic gastric or GEJ adenocarcinoma.	NCT06346392
	Esophageal Squamous Cell Carcinoma	Ifinatamab Deruxtecan (I-DXd)	B7-H3	To evaluate the efficacy and safety of I-DXd compared to investigator’s choice of chemotherapy in patients with previously treated advanced or metastatic esophageal squamous cell carcinoma.	NCT06644781
Gynecological Tumors	Platinum-Resistant Recurrent Epithelial Ovarian Cancer	HS-20089	B7-H4	To evaluate the efficacy and safety of HS-20089 compared to investigator’s choice of chemotherapy in patients with platinum-resistant recurrent epithelial ovarian cancer.	NCT06855069
	Platinum-Resistant Ovarian Cancer, Fallopian Tube Cancer, Primary Peritoneal Cancer	Raludotatug Deruxtecan (R-DXd)	CDH6	To evaluate the safety and efficacy of R-DXd in patients with platinum-resistant high-grade ovarian, fallopian tube, or primary peritoneal cancer.	NCT06161025
	FOLR1-Positive Ovarian Cancer	Luveltamab tazevibulin	FOLR1	To investigate the efficacy and safety of Luveltamab tazevibulin compared to investigator’s choice of chemotherapy in patients with FOLR1-positive ovarian cancer (including fallopian tube or primary peritoneal cancer).	NCT05870748
	Cervical Cancer	Tisotumab vedotin	TF	To compare the efficacy of Tisotumab vedotin with chemotherapy in patients with recurrent or metastatic cervical cancer.	NCT04697628
Urinary System Tumors	HER2-Positive Urothelial Carcinoma	MRG002	HER2	To compare the overall survival and progression-free survival of MRG002 with investigator’s choice of chemotherapy in patients with HER2-positive unresectable advanced or metastatic urothelial carcinoma.	NCT05754853
	Urothelial Carcinoma	Sacituzumab Govitecan-hziy	TROP2	To compare the overall survival of Sacituzumab govitecan with physician’s choice of treatment in patients with metastatic or locally advanced unresectable urothelial carcinoma.	NCT04527991
	Urothelial Carcinoma	Enfortumab vedotin	Nectin-4	To compare the efficacy of Enfortumab vedotin combined with pembrolizumab versus standard chemotherapy in patients with metastatic urothelial carcinoma.	NCT04223856
Hematological Tumors	Diffuse Large B-Cell Lymphoma	Zilovertamab vedotin	ROR1	To evaluate the efficacy (progression-free survival) of Zilovertamab vedotin combined with R-CHP versus R-CHOP in previously untreated DLBCL patients.	NCT06717347
	Relapsed/Refractory Diffuse Large B-Cell Lymphoma	Zilovertamab vedotin	ROR1	To evaluate the safety and efficacy (progression-free survival) of Zilovertamab vedotin combined with standard therapy (R-GemOx) versus standard therapy in patients with rrDLBCL.	NCT05139017
	Relapsed/Refractory Diffuse Large B-Cell Lymphoma	Loncastuximab tesirine	CD19	To compare the efficacy of Loncastuximab tesirine combined with rituximab versus standard immunochemotherapy in patients with relapsed/refractory DLBCL.	NCT04384484
	Diffuse Large B-Cell Lymphoma	Polatuzumab vedotin	CD79b	To compare the efficacy, safety, and pharmacokinetics of Polatuzumab vedotin combined with R-CHP versus R-CHOP in previously untreated DLBCL patients.	NCT03274492
	Hodgkin Lymphoma	BV	CD30	To compare the modified progression-free survival of BV combined with AVD versus ABVD as first-line treatment in advanced classical Hodgkin lymphoma.	NCT01712490
	Multiple Myeloma	Belantamab mafodotin	BCMA	To evaluate the effect of Belantamab mafodotin combined with other anticancer drugs in patients with relapsed/refractory multiple myeloma.	NCT04126200
Other Solid Tumors	Head and Neck Squamous Cell Carcinoma	MRG003	EGFR	To compare the efficacy and safety of MRG003 versus cetuximab/methotrexate as second/third-line therapy in patients with recurrent metastatic head and neck squamous cell carcinoma.	NCT05751512
	Osteosarcoma	HS-20093	B7-H3	To evaluate the efficacy and safety of HS-20093 compared to gemcitabine combined with docetaxel in patients with osteosarcoma who have failed at least two lines of therapy.	NCT06935409
	Glioblastoma	Depatuxizumab mafodotin (ABT-414)	EGFR	To evaluate the overall survival of ABT-414 combined with radiotherapy and temozolomide followed by ABT-414 combined with adjuvant temozolomide in patients with newly diagnosed EGFR-amplified glioblastoma.	NCT02573324

Abbreviations: ADC: antibody–drug conjugate; HER2: human epidermal growth factor receptor 2; HR: hormone receptor; PFS: progression-free survival; T-DM1: trastuzumab emtansine; TCBHP: trastuzumab + pertuzumab + docetaxel + carboplatin; Dato-DXd: datopotamab deruxtecan; T-DXd: trastuzumab deruxtecan; I-DXd: ifinatamab deruxtecan; EGFR: epidermal growth factor receptor; GEJ: gastroesophageal junction; R-CHP: rituximab + cyclophosphamide + doxorubicin + prednisone; R-CHOP: rituximab + cyclophosphamide + doxorubicin + vincristine + prednisone; DLBCL: diffuse large B-Cell lymphoma; rrDLBCL: Relapsed/Refractory Diffuse Large B-Cell Lymphoma; R-GemOx: rituximab + gemcitabine + oxaliplatin; AVD: doxorubicin + vinblastine + dacarbazine; ABVD: doxorubicin + bleomycin + vinblastine + dacarbazine.

**Table 3:** Current research on next-generation ADCs.

Drug/Agent	Target(s)	Mechanism/Innovation Point	Current Research Stage/Key Findings	Reference
BL-B01D1	EGFR x HER3	Bispecific ADC targeting both EGFR and HER3; payload is a novel TOP1 inhibitor.	Phase I (NCT05194982); Shows high ORR (52.5–63.5%) in heavily pretreated EGFR-mutant NSCLC and other solid tumors; manageable toxicity.	[214]
BDC-1001	HER2	ISAC carrying a TLR7/8 agonist; designed to activate innate immunity in situ.	Phase I/II (NCT04278144); Shows limited monotherapy efficacy in HER2-positive solid tumors, with some patients achieving stable disease; highlights challenges in safely activating immunity with ISACs.	[244]
XMT-2056	HER2	ISAC carrying a STING agonist; designed to activate the STING pathway in both tumor cells and tumor-resident immune cells.	Phase I (previously paused, resumed); Preclinical studies show induction of anti-tumor immunity by acting on both cancer cells and immune cells.	[230]
ORM-5029	HER2	DAC carrying a GSPT1 protein degrader (PROTAC); degrades target protein via the ubiquitin-proteasome system.	Phase I (NCT05511844); In patients with HER2-expressing advanced solid tumors; Preclinical studies show anti-tumor activity comparable to T-DXd in both high and low HER2 expression models.	[233]
CX-2009 (Pralvaztamab Ravtansine)	CD166	Probody™ ADC; antibody masked by a peptide that is cleaved by proteases in the tumor microenvironment to activate.	Phase I/II; Evaluated in advanced solid tumors, but efficacy and toxicity control did not fully meet expectations, illustrating challenges of Probody ADC technology.	[217]
ARX788	HER2	Highly stable ADC utilizing non-natural amino acid incorporation for site-specific conjugation; non-cleavable linker.	Phase I; Shows anti-tumor activity in HER2-positive metastatic breast cancer, but target-related toxicities (ocular) were observed.	[197]
DCDS0780A	CD79b	Site-specific ADC utilizing engineered cysteines for conjugation.	Phase I; Evaluated in B-cell non-Hodgkin lymphoma; development hampered due to significant toxicities (ocular).	[198]
ADDIN EN.CITE.DATA ADDIN EN.CITE Enfortumab Vedotin	Nectin-4	Traditional ADC; resistance research associated with target downregulation, prompting exploration of new strategies to overcome resistance.	Approved for urothelial carcinoma; Studies find frequent decrease in membranous Nectin-4 expression in metastases, associated with resistance.	[190]
Cetuximab Saratolacan (RM-1929)	EGFR	Photoimmunotherapy (PIT); antibody Cetuximab conjugated to photosensitizer IR700; cell killing activated by NIR light.	Approved in Japan for locally advanced or recurrent head and neck squamous cell carcinoma; suitable for superficial tumors, applied via light-guided fiber endoscopy.	[225]
GPC2-D3-PBD ADC	GPC2	Immunomodulatory ADC; payload is PBD dimer; directly kills tumor cells and induces ICD, remodeling the tumor immune microenvironment.	Preclinical (neuroblastoma); shows synergy when combined with immunomodulators.	[231]
^177^Lu-PSMA-617 (Pluvicto^®^)	PSMA	Radioligand Therapy/RDC; payload is radioactive Lutetium-177 (^177^Lu); killing is internalization-independent and exhibits a crossfire effect.	Approved for PSMA-positive metastatic castration-resistant prostate cancer; effectively overcomes tumor heterogeneity.	[234]
Anti-B7-H3 Dual-Payload ADC	CD276/B7-H3	Dual-payload ADC co-conjugated with a tubulin inhibitor and a TOP1 inhibitor; combines direct cytotoxicity with immune activation.	Preclinical (triple-negative breast cancer); exhibits potent cytotoxicity and immune activation, with efficacy superior to single-payload ADCs.	[239]
Antibody-DNA Nanostructure ADC	Not Specified	Nanotechnology-based ADC; uses DNA nanotechnology to build a programmable nano-platform with high drug loading capacity; enhances binding and endocytosis to cancer cells.	Preclinical; achieves efficient drug delivery and controlled release, reducing toxicity to normal cells.	[240]
HER2-Targeted Polymeric Micelle ADC	HER2	Nanotechnology-based ADC; conjugates Trastuzumab to PEG-polyhistidine copolymer micelles, improving drug delivery.	Preclinical (breast cancer models) shows improved tumor cell selectivity and enhanced intracellular delivery efficiency.	[241]
T-DM1-IR700 (Example)	HER2	Photoactivatable ADC; T-DM1 conjugated to photosensitizer IR700; NIR light triggers membrane rupture and drug release.	Preclinical; NIR light-induced drug release and cytotoxicity confirmed in xenograft models, generating a “photo-bystander effect”.	[63]
Hypoxia-Responsive ADC (Example)	Not Specified	Smart linker ADC; uses an azobenzene linker that is cleaved by reduction in the hypoxic tumor microenvironment, releasing the payload.	Preclinical; demonstrates selective activation under hypoxic conditions, effectively reducing off-target toxicity and improving the therapeutic window.	[221]
Bioorthogonal ‘Click-Release’ ADC (Example)	Not Specified	Bioorthogonal chemistry-controlled release ADC; payload release triggered by exogenous administration of a trigger molecule (tetrazine) which undergoes a click reaction with trans-cyclooctene on the ADC.	Preclinical; achieves on-demand, localized activation of ADC cytotoxicity in mouse models, demonstrating potential for theranostic applications (imaging to therapy).	[245]
Dual-Payload ADC via AJICAP Technology	HER2	Dual-payload ADC utilizing AJICAP site-specific conjugation technology to precisely load two drugs with different mechanisms onto the same antibody.	Preclinical; demonstrates excellent in vitro and in vivo anti-tumor efficacy in HER2-positive tumor models, with good physicochemical stability.	[236]
SPARC Platform Multi-Payload ADC	Not Specified	Multi-payload ADC architecture; allows programmable combination of different mechanism drugs on a single antibody for synergistic release.	Preclinical; achieves synergistic drug release in vivo through co-delivery of different drugs, reducing off-target toxicity and enhancing tumor killing.	[44]

Abbreviations: ADC: antibody–drug conjugate; DAC: degrader–antibody conjugate; EGFR: epidermal growth factor receptor; HER: human epidermal growth factor receptor; ISAC: immune-stimulating antibody conjugate; NSCLC: non-small cell lung cancer; ORR: objective response rate; PBD: pyrrolobenzodiazepine; PIT: photoimmunotherapy; PROTAC: PROteolysis TArgeting Chimera; PSMA: prostate-specific membrane antigen; RDC: radiopharmaceutical drug conjugate; STING: stimulator of interferon genes; TLR: toll-like receptor; TOP1: topoisomerase I; ICD: immunogenic cell death; NIR: near-infrared.

**Table 4:** Clinically validated versus emerging ADC platforms.

**Platform Category**		**Core Design**	**Example (Drug Candidate)**	**Clinical Validation Stage**	**Key Features**	**Reference**
Clinically Validated Platforms	Cytotoxic ADC (Standard)	Monoclonal antibody + cytotoxic payload via cleavable/non-cleavable linker.	Trastuzumab emtansine (T-DM1), Brentuximab vedotin (BV)	Approved; Standard of care in multiple indications.	Foundation of ADC field; “Targeted chemotherapy”; Bystander effect (cleavable linker).	[17]
	Site-Specific Conjugation ADC	Engineered antibodies (e.g., THIOMAB, unnatural amino acids) for homogeneous Drug-to-Antibody Ratio (DAR).	Trastuzumab deruxtecan (T-DXd, DS-8201)	Approved; Demonstrated superior efficacy in several trials.	Improved pharmacokinetics, therapeutic index, and manufacturability; Enables higher DAR.	[38]
	Bispecific ADC (BsADC)	Bispecific antibody targeting two tumor-associated antigens, conjugated to a cytotoxic payload.	BL-B01D1 (EGFRxHER3)	Phase I/II (Emerging, but platform clinically validated in trials).	Enhanced tumor selectivity, internalization; Potential to overcome antigen heterogeneity.	[214]
	Trop-2 Directed ADC	Targeting TROP2, a pan-epithelial antigen, with topoisomerase I inhibitor payload.	Sacituzumab govitecan (SG, Trodelvy)	Approved for TNBC and HR+/HER2- breast cancer.	Expanded ADC utility to “non-HER2” solid tumors; Durable responses in refractory settings.	[147]
Emerging Platforms	Immune-Stimulating ADC (ISAC)	Replaces cytotoxic payload with immune agonist (e.g., TLR, STING agonist) to remodel tumor microenvironment.	BDC-1001 (TLR7/8), XMT-2056 (STING)	Phase I/II; Faced challenges with narrow therapeutic window.	Aims to convert “cold” to “hot” tumors; Potential synergy with ICIs; Toxicity management is key.	[229]
	Degrader-Antibody Conjugate (DAC)	Payload is a PROTAC or molecular glue, inducing targeted protein degradation via ubiquitin-proteasome system.	ORM-5029 (HER2-targeted GSPT1 degrader)	Phase I.	Potential to target “undruggable” proteins; Novel mechanism beyond cytotoxicity.	[233]
	Radiopharmaceutical Drug Conjugate (RDC)	Antibody conjugated to a therapeutic radionuclide (e.g., ^177^Lu).	[^177^Lu]Lu-PSMA-617 (Pluvicto)	Approved for prostate cancer; Platform validated.	Crossfire effect kills neighboring cells; Action independent of internalization; Theranostic potential.	[234]
	Dual-/Multi-Payload ADC	Single antibody conjugated to two or more distinct payloads with different mechanisms of action.	Anti-B7-H3 dual-payload ADC (Tubulin inhibitor + TOP1 inhibitor)	Preclinical/Early Clinical.	Aims to overcome heterogeneity and prevent resistance via synergistic killing; Requires advanced conjugation tech.	[238]
	Probody^®^-ADC (Masked ADC)	Antibody binding site masked by a peptide substrate cleaved by tumor-specific proteases.	CX-2009 (Praluzatamab ravtansine, anti-CD166)	Phase I/II (Development challenged).	Designed to minimize “on-target, off-tumor” toxicity by limiting activation to tumor microenvironment.	[217]
	Photoactivatable/Smart-Responsive ADC	Linker cleavage triggered by external (e.g., near-infrared light) or internal (e.g., hypoxia, high ROS) stimuli.	Cetuximab saratolacan (RM-1929, PIT)	Approved in Japan for head and neck cancer (PIT).	Unprecedented spatiotemporal control; Reduces systemic toxicity; Suitable for superficial/localized tumors.	[225]

## Data Availability

No new data were created or analyzed in this study. Data sharing is not applicable to this article.

## Glossary

**Abbreviation**	**Full Name**
A + AVD	Brentuximab vedotin + doxorubicin + vinblastine + dacarbazine
ABVD	Doxorubicin + bleomycin + vinblastine + dacarbazine
ABC	ATP-binding cassette
ADCs	Antibody–drug conjugates
ALL	Acute lymphoblastic leukemia
AML	Acute myeloid leukemia
ASCT	Autologous hematopoietic stem cell transplantation
ATP	Adenosine triphosphate
B-ALL	B-cell acute lymphoblastic leukemia
BCMA	B-cell maturation antigen (TNFRSF17)
BsADC	Bispecific antibody–drug conjugate
BV	Brentuximab vedotin
cHL	Classical Hodgkin lymphoma
DAC	Degrader–antibody conjugate
DAR	Drug-to-antibody ratio
Dato-DXd	Datopotamab deruxtecan
DLBCL	Diffuse large B-cell lymphoma
DOR	Duration of response
EV	Enfortumab vedotin
FDA	U.S. Food and Drug Administration
FRα	Folate receptor-alpha
HCL	Hairy cell leukemia
HER	Human epidermal growth factor receptor
HMGB1	High-mobility group box 1
HR	Hazard ratio
HSCT	Hematopoietic stem cell transplantation
ICI	Immune checkpoint inhibitor
ICD	Immunogenic cell death
IHC	Immunohistochemistry
ILD	Interstitial lung disease
ISAC	Immune-stimulating antibody conjugate
LBCL	Large B-cell lymphoma
MMAE	Monomethyl auristatin E
NIR	Near-infrared
NSCLC	Non-small cell lung cancer
ORR	Objective response rate
PBD	Pyrrolobenzodiazepine
PD-1/PD-L1	Programmed death-1/ligand-1
PEG	Polyethylene glycol
PFS	Progression-free survival
PIT	Photoimmunotherapy
Pola	Polatuzumab vedotin
PROs	Patient-reported outcomes
PROTAC	PROteolysis-TArgeting Chimera
PSMA	Prostate-specific membrane antigen
R-CHOP	Rituximab + cyclophosphamide + doxorubicin + vincristine + prednisone
R-CHP	Rituximab + cyclophosphamide + doxorubicin + prednisone
RAB4a	Ras-related protein Rab-4A
RAB5A	Ras-related protein Rab-5A
RDC	Radionuclide (radiopharmaceutical) drug conjugate
ROS	Reactive oxygen species
SPARC	Synchronized payload release conjugate
SSTR	Somatostatin receptor
STING	Stimulator of interferon genes
T-DXd	Trastuzumab deruxtecan
TCE/TCR	Triple-class exposed/triple-class refractory
TF	Tissue factor
TLR	Toll-like receptor
TME	Tumor microenvironment
TOP1	Topoisomerase I
TROP2	Trophoblast cell surface antigen 2 (TACSTD2)
TV	Tisotumab vedotin
UC	Urothelial carcinoma
Val-Cit	Valine–citrulline
VOD	Veno-occlusive disease
